# mTOR: a link from the extracellular milieu to transcriptional regulation of oligodendrocyte development

**DOI:** 10.1042/AN20120092

**Published:** 2013-03-19

**Authors:** Teresa L. Wood, Kathryn K. Bercury, Stacey E. Cifelli, Lauren E. Mursch, Jungsoo Min, Jinxiang Dai, Wendy B. Macklin

**Affiliations:** *Department of Neurology and Neuroscience, University of Medicine and Dentistry of New Jersey, Newark, NJ, U.S.A.; †Department of Cell and Developmental Biology, University of Colorado School of Medicine, Aurora, CO, U.S.A.

**Keywords:** mammalian target of rapamycin (mTOR), mTOR complex 1 (mTORC1), mTOR complex 2 (mTORC2), myelin, myelination, oligodendrocyte, 4EBP1-3, eIF4E-binding proteins, AGC-type kinases, protein kinase A/protein kinase G/protein kinase C-family kinases, bHLH, basic helix–loop–helix, BMP, bone morphogenetic protein, C/EBP, CCAAT/enhancer-binding protein, CNS, central nervous system, ERK, extracellular signal-regulated kinase, FKBP12, FK506-binding protein 12, GalC, galactosyl cerebroside, GPR17, G-protein coupled receptor 17, HAT, histone acetyl transferase, HDAC, histone deacetylase, HIF1α, hypoxia-induced factor 1α, ID, inhibitor of DNA binding/differentiation, IGF-I, insulin-like growth factor 1, IGF-IR, IGF type 1 receptor, IRS, insulin receptor substrate, MAPK, mitogen-activated protein kinase, MBP, myelin basic protein, mSIN1, mammalian stress-activated protein kinase interacting protein 1, mTOR, mammalian target of rapamycin, mTORC1, raptor-mTOR complex, mTORC2, rictor-mTOR complex, OPC, oligodendrocyte progenitor cell, PDGF, platelet-derived growth factor, PDK2, phosphoinositide-dependent kinase 2, PGC-1α, peroxisome-proliferator-activated receptor coactivator-1α, PI3K, phosphoinositide 3-kinase, PKC-α, protein kinase C-α, PLP, proteolipid protein, PNS, peripheral nervous system, PPARγ, peroxisome-proliferator-activated-receptor γ, PRAS40, praline-rich Akt substrate of 40 kDa, protor, protein observed with rictor, PTEN, phosphatase and tensin homologue deleted on chromosome 10, Rheb, Ras homologue enriched in brain, S6K1/2, S6 kinase, SGK1, serum- and glucocorticoid-induced protein kinase 1, siRNA, small interfering RNA, SREBP-1, sterol-regulatory-element-binding protein, TCF4/TCF7L2, T cell factor 4, TSC, tuberous sclerosis complex, VEGF, vascular endothelial growth factor, YY1, Yin Yang 1

## Abstract

Oligodendrocyte development is controlled by numerous extracellular signals that regulate a series of transcription factors that promote the differentiation of oligodendrocyte progenitor cells to myelinating cells in the central nervous system. A major element of this regulatory system that has only recently been studied is the intracellular signalling from surface receptors to transcription factors to down-regulate inhibitors and up-regulate inducers of oligodendrocyte differentiation and myelination. The current review focuses on one such pathway: the mTOR (mammalian target of rapamycin) pathway, which integrates signals in many cell systems and induces cell responses including cell proliferation and cell differentiation. This review describes the known functions of mTOR as they relate to oligodendrocyte development, and its recently discovered impact on oligodendrocyte differentiation and myelination. A potential model for its role in oligodendrocyte development is proposed.

## INTRODUCTION

CNS (central nervous system) myelin is produced by the differentiation of the oligodendroglial plasma membrane, which surrounds axons in a compact multilamellar structure acting both to insulate axons and facilitate nerve impulse conduction as well as to provide trophic support for the axon. Oligodendrocyte development has been extensively characterized, and in early studies a number of extracellular signals including several growth factors were identified that influence OPC (oligodendrocyte progenitor cell) survival, proliferation and differentiation (for overviews; see Miller, 2002; Baron et al., 2005).

Oligodendrocyte lineage development is defined by distinct morphology changes both *in vitro* and *in vivo* (Pfeiffer et al., 1993; Song et al., 2001), which is achieved by rapid gene expression and cytoskeletal changes in response to extracellular signals (Song et al., 2001; Liang et al., 2004; Lafrenaye and Fuss, 2010; Rajasekharan et al., 2010; Colognato and Tzvetanova, 2011; Eyermann et al., 2012). In addition to investigations of extracellular signals, numerous studies have focused on identifying the transcription factors that regulate oligodendrocyte specification and differentiation (for overviews, see Wegner, 2008; Li et al., 2009; Emery, 2010a, 2010b). These studies have highlighted the importance of 10–15 such factors, and extensive data exist demonstrating the complex regulation of gene expression at different stages of oligodendrocyte development (Wegner, 2008; Li et al., 2009).

An understudied research area until recently has been the intracellular signalling pathways that link these two essential regulators of oligodendrocyte development: surface receptor signalling and the transcription factors that regulate oligodendrocyte-specific gene expression. This review focuses on one such pathway: the mTOR (mammalian target of rapamycin) pathway, which is downstream of PI3K (phosphoinositide 3-kinase)/Akt, and which regulates many aspects of cell development. The PI3K/Akt pathway is pivotal in many survival and growth factor signalling systems (Franke et al., 1995; Dudek et al., 1997; Franke et al., 1997; Kennedy et al., 1997), where it acts through phosphorylation of numerous substrates that promote cell survival and proliferation. PI3K/Akt signalling mediates oligodendrocyte progenitor cell survival/proliferation induced by PDGF (platelet-derived growth factor) (Ebner et al., 2000; Baron et al., 2003) and IGF-I (insulin-like growth factor 1) (Ness et al., 2002; Ness and Wood, 2002; Ness et al., 2004; Zaka et al., 2005; Cui and Almazan, 2007; Frederick et al., 2007; Min et al., 2012). Of particular interest for this review, is the role of PI3K/Akt signalling in cell differentiation reported in many cell systems (Fishwick et al., 2010; Baracho et al., 2011; Gardner et al., 2012). In particular, recent studies have shown that the PI3K/Akt pathway, through mTOR, promotes oligodendrocyte differentiation and myelination in the CNS and in the PNS (peripheral nervous system) (Narayanan et al., 2009; Tyler et al., 2009; Tyler et al., 2011; Guardiola-Diaz et al., 2012; Sherman et al., 2012).

In the following sections, we provide an overview of mTOR signalling complexes and targets, their known functions in translation regulation, transcription, RNA processing and cell differentiation in non-myelin producing cells. We then discuss our current knowledge of mTOR signalling in oligodendrocyte development and in developmental myelination in the CNS and PNS as a downstream mediator of PI3K/Akt signalling. Finally, we connect mTOR signalling with downstream transcriptional regulation of oligodendrocyte differentiation and myelination programmes, and we provide perspectives on how mTOR probably links extracellular and intracellular mediators and coordinates with other signalling pathways to promote a fully differentiated myelinating phenotype in oligodendrocytes.

## THE MAMMALIAN TARGET OF RAPAMYCIN (mTOR)

**Figure 1:**
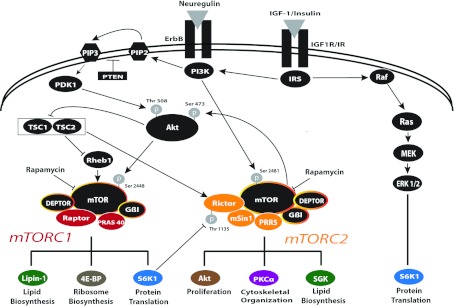
mTOR signalling pathways Model of the growth factor-mediated regulation of mTOR signalling through mTORC1 and mTORC2. The well-established function of mTOR in nutrient sensing is not described here. Extracellular factors, such as IGF-I, insulin or neuregulin, bind to their respective receptors [IGF-IR, insulin receptor (IR) or ErbBs] and activate the PI3K/Akt pathway, which targets mTORC1 and mTORC2 to regulate unique cellular processes. PI3K activates PDK1, which phosphorylates Akt on Thr^308^. This activates mTORC1 directly or indirectly through inactivation of TSC1/2 to eliminate its inactivation of Rheb1, thereby activating mTORC1. mTORC2 can be directly activated by PI3K or TSC1/2 and is inactivated by S6K1, a target of mTORC1 and of the Erk 1/2 pathway, which is also activated by these factors. See text for details on these interactions. Yellow-encircled black symbols represent conserved proteins between mTORC1 and mTORC2. Arrows indicate positive interactions; bars indicate negative interactions.

mTOR is a highly evolutionarily conserved serine/threonine protein kinase, which is a downstream mediator of PI3K/Akt signalling in organisms from yeast to mammals. mTOR is ubiquitously expressed in cells, and it regulates multiple cellular functions including survival, proliferation, organogenesis and differentiation of numerous cell types (Hwang et al., 2008). Canonical signalling through mTOR has been widely studied in autophagy, where mTOR acts as an amino acid sensor responding to cellular stress and as the hub for integration of extracellular signals to regulate cell growth (for reviews, see Sarbassov et al., 2005a; Dann and Thomas, 2006; Wullschleger et al., 2006). mTOR is essential for development; mTOR-null mice die during early embryogenesis (Guertin et al., 2006b). Growth factors such as insulin or IGF-I activate mTOR signalling by activating PI3K/Akt. Activated Akt inhibits TSC (tuberous sclerosis complex) 1/2, thereby relieving its inhibition of Rheb (Ras homologue enriched in brain), allowing Rheb activation of mTOR (Figure 1; for review, see Avruch et al., 2006). mTOR is phosphorylated at Ser^2448^ by PI3K/Akt signalling and is autophosphorylated at Ser^2481^(Sekulic et al., 2000; Soliman et al., 2010); Ser^2481^ autophosphorylation is required for its activity (Soliman et al., 2010).

### mTOR complexes and targets

mTOR exists in two functionally distinct complexes, the mTORC1 (raptor–mTOR complex) and the mTORC2 (rictor–mTOR complex). The mTORC1 complex is the better studied of the two complexes because it regulates translation of 5′ TOP (terminal oligopyrimidine tract)-containing mRNAs in response to extracellular signals (Huo et al., 2011), and it is central to a signalling cascade that regulates initiation of cap-dependent RNA translation. (Cap-independent translation is unaffected by mTOR signalling.) 4EBP1–3 (eIF4E-binding proteins 1–3) bind to the mRNA cap-binding protein eIF4E, inhibiting the initiation of cap-dependent translation. mTOR phosphorylates 4EBP1–3, which inhibits their activity and consequently allows release of eIF4E to promote cap-dependent translation. Additionally, the p70 ribosomal proteins S6K (S6 kinase) 1/2 that phosphorylate the S6 protein of small ribosomal subunits are also phosphorylated and activated by mTORC1, along with other kinases, thereby enhancing protein translation.

In comparison with mTORC1, the biological function of mTORC2 is less well understood, in part because of its more recent identification (Sarbassov et al., 2005b). mTORC2 can be activated by PI3K (Dalle Pezze et al., 2012) or by TSC1/2 (Huang et al., 2008) or inactivated by mTORC1-activated S6K1 (Dibble et al., 2009; Julien et al., 2010) (Figure 1). mTORC2, also known as PDK2 (phosphoinositide-dependent protein kinase-2), phosphorylates the hydrophobic motif of Akt at Ser^473^, and of closely related AGC-type kinases (protein kinase A/protein kinase G/protein kinase C-family kinases) (Parekh et al., 1999; Sarbassov et al., 2005b; Guertin et al., 2006b). The knockout of mTORC2-specific rictor is embryonic lethal and results in complete loss of Ser^473^ phosphorylation of Akt (Yang et al., 2006a), which is essential for the maximal activation of Akt. In contrast, mTORC1 does not phosphorylate Akt. Interestingly, mTORC2 knockdown has no effect on S6K1 or 4EBP (enhancer-binding protein) phosphorylation, suggesting that mTORC2 targets an Akt pool that is distinct from the Akt pool upstream of mTORC1 (Jacinto et al., 2004; Sarbassov et al., 2004). In some cell types, mTORC2 has a major role in the organization of the cytoskeleton (Jacinto et al., 2004; Sarbassov et al., 2004; Liu et al., 2008), mediated by its phosphorylation of PKC-α (protein kinase C-α) (Jacinto et al., 2004; Guertin et al., 2006b). Additionally, mTORC2 regulates SGK1 (serum- and glucocorticoid-induced protein kinase 1) activity, a major regulator of Forkhead transcription factors and of MEKK2 [MAPK (mitogen-activated protein kinase)/ERK (extracellular signal-regulated kinase) kinase 2], which impacts the MAPK (mitogen-activated protein kinase) pathways (Guertin et al., 2006a; Pearce et al., 2010).

The multimeric mTORC1 contains mTOR, raptor and several other adaptor proteins, including mLST8 (mammalian lethal with Sec13 protein 8) and Deptor, which are also shared with mTORC2 (Hara et al., 2002; Kim et al., 2002; Kim et al., 2003) (Figure 1). By contrast, the negative regulator PRAS40 (praline-rich Akt substrate of 40 kDa) is unique to mTORC1 (Kovacina et al., 2003; Vander Haar et al., 2007). PRAS40 negatively regulates mTORC1 by binding to raptor and inhibiting the recruitment of other mTORC1 substrates critical for protein translation; upon insulin stimulation, PRAS40 is phosphorylated, releasing its inhibition of mTORC1 activity (Sancak et al., 2007).

Proteins unique to mTORC2 include protor (protein observed with rictor) (Pearce et al., 2007). Protor-null mice have significantly reduced SGK1 phosphorylation (Pearce et al., 2011), consistent with the data discussed above that SGK1 is an mTORC2 target. mSIN1 (mammalian stress-activated protein kinase interacting protein 1) is also unique to mTORC2. It has recently been shown that mSin1 phosphorylation prevents the lysosomal degradation of mTORC2 (Chen and Sarbassov, 2011).

*In vivo*, mTOR^fl/fl^ (Risson et al., 2009; Lang et al., 2010), raptor^fl/fl^ (Sengupta et al., 2010) and rictor^fl/fl^ (Kumar et al., 2008) mice have been created to establish conditional knockouts to study the selective loss of mTOR or of each complex in different tissues and cell types. Studies using these mouse lines are rapidly emerging and include loss of mTOR in muscle (Risson et al., 2009; Lang et al., 2010) or Schwann cells (Sherman et al., 2012), of raptor in liver (Sengupta et al., 2010), thymocytes (Tang et al., 2012) or the haematopoietic lineage (Hoshii et al., 2012; Kalaitzidis et al., 2012) and of rictor in muscle (Kumar et al., 2008), prostate (Guertin et al., 2009), fat (Kumar et al., 2010), neurons (Siuta et al., 2010), beta cells (Gu et al., 2010), T-cells/thymocytes (Lee et al., 2010; Tang et al., 2012), liver (Yuan et al., 2012), the haematopoietic lineage (Kalaitzidis et al., 2012) or neural progenitor cells (Carson et al., 2013). These studies demonstrate the wide importance for the mTOR complexes in many cell systems.

#### mTOR inhibitors

Rapamycin is a specific inhibitor of mTOR (Dann and Thomas, 2006; Wullschleger et al., 2006). It inhibits mTOR by binding with high affinity to its internal receptor, FKBP12 [FK506-binding protein 12 (FK506 is an immunosuppressant macrolide)]. When rapamycin-bound FKBP12 binds to free mTOR at the FKBP12-binding domain, there is allosteric hinderance that blocks raptor binding to mTOR. FKBP12–rapamycin inhibits mTORC1 activity both by disrupting mTOR–raptor association and by inhibiting mTOR autophosphorylation, but rapamycin does not inhibit all mTOR function. mTORC2 was originally thought to be rapamycin-insensitive, since it does not interact with FKBP12–rapamycin (Sarbassov et al., 2004). However, high doses or prolonged exposure to rapamycin inhibit mTORC2 assembly by sequestering free mTOR so it is no longer available to form mTORC2, and Akt Ser^473^ phosphorylation is no longer maintained (Sarbassov et al., 2006).

Several mTOR inhibitors have been developed recently, including Torin 1, a competitive ATP inhibitor, which fully blocks both mTORC1 and mTORC2 (Thoreen et al., 2009; Liu et al., 2010; Liu et al., 2012). However, there has yet to be an inhibitor that specifically blocks only mTORC2, despite emerging data that mTORC2 may be a very important cancer target (Sparks and Guertin, 2010).

### mTOR function: more than translation

While it is widely known that mTORC1 promotes mRNA translation in response to extracellular signals, there is evidence that mTOR is also involved in transcriptional regulation and RNA processing (Hannan et al., 2003; Cunningham et al., 2007; Willis and Moir, 2007; Kantidakis et al., 2010; Shor et al., 2010). An example of its role in RNA processing is the splicing factor SF2/ASF, which interacts with mTOR as a scaffold on specific mRNAs to regulate RNA splicing, stability, nuclear export and promote translation initiation (Michlewski et al., 2008; White et al., 2010).

mTOR signalling regulates many genes in metabolic and biosynthetic pathways in mammalian cells (Peng et al., 2002; Duvel et al., 2010). In particular, mTORC1, the complex that is more responsive to metabolic conditions, upregulates genes controlling protein and nucleotide biosynthesis, lipid/sterol biosynthesis, mitochondrial and glycolytic metabolism, and the pentose phosphate shunt, while down-regulating genes controlling general catabolism. In skeletal muscle cells, mTORC1 acts as a nutrient sensor to maintain mitochondrial energy production by increasing transcription of genes that encode oxidative metabolism complex proteins (Cunningham et al., 2007).

**Figure 2:**
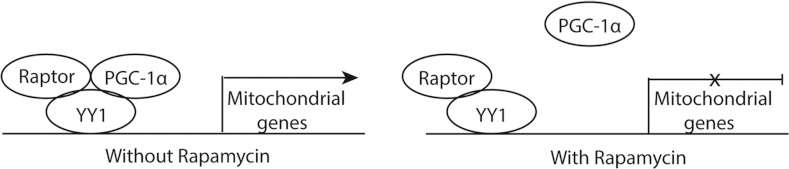
Interaction of transcription factors and raptor to regulate mitochondrial gene expression In skeletal muscle cells, PGC-1α, YY1 and raptor form a complex in the nucleus to activate mitochondrial gene expression. Upon rapamycin (an mTOR inhibitor) treatment, PGC-1α no longer complexes with YY1, inhibiting mitochondria gene transcription. Adapted and reprinted by permission from Macmillan Publishers Ltd: Cunningham JT, Rodgers JT, Arlow DH, Vazquez F, Mootha VK, Puigserver P (2007) mTOR controls mitochondrial oxidative function through a YY1-PGC-1 α transcriptional complex. Nature 450:736-740, copyright 2007.

Regulation of mitochondrial genes in muscle is a particularly relevant example for transcriptional regulation by mTOR as it involves the interaction between raptor, the unique mTORC1 protein and the zinc-finger transcription factor YY1 (Yin Yang 1) (Cunningham et al., 2007), which also is important in oligodendrocyte differentiation (see later sections). In skeletal muscle, the transcriptional regulator PGC-1α (peroxisome-proliferator-activated receptor coactivator 1α) functions as a coactivator of YY1 in an mTOR-dependent manner, such that raptor forms a complex with YY1 and PGC-1α to bind mitochondrial gene promoters and enhance transcription (Figure 2). Following rapamycin treatment and consequent mTORC1 inhibition, PGC-1α can no longer associate with YY1 to promote mitochondrial gene transcription (Cunningham et al., 2007).

In addition to regulating metabolic processes in normal cells, mTORC1 regulates several transcription factors that control gene expression during cell stress such as hypoxia (Dunlop and Tee, 2009; Duvel et al., 2010). mTOR is a positive regulator of HIF-1α (hypoxia-induced factor1α) activation by hypoxia in cancer cells (Hudson et al., 2002). The HIF complex regulates hypoxia-inducible genes such as erythropoietin and VEGF (vascular endothelial growth factor). The anti-cancer activity of rapamycin *in vivo* may result from inhibition of the hypoxia response programme in developing tumours.

The impact of mTORC2 on transcription is less well understood. In some cell systems, mTORC1 is predominantly cytoplasmically localized, whereas mTORC2 is found in both the nucleus and cytoplasm, suggesting a nuclear role for mTORC2 (Rosner and Hengstschlager, 2008). Rapamycin causes the dephosphorylation of the mTORC2 proteins, mSin1 and rictor and their translocation out of the nucleus (Rosner and Hengstschlager, 2008). Many aspects of mTOR regulation of transcription, including whether one or both mTOR complexes are involved, are still unclear. Nevertheless, it is clear that mTOR has a unique role in regulating the activity of specific transcription factors and genes that regulate multiple cellular cascades.

### Cell differentiation and TOR

Most functional studies on the PI3K/Akt/TOR pathway have focused on its role in cell size and proliferation, the latter particularly in transformed cells. However, several investigators have reported a role for this pathway in differentiation of specific cell types, which is most relevant to its proposed role in regulating the myelination programme. Thus, we briefly review literature on mTOR function in cell differentiation in other systems including its described role in lipid synthesis in adipocytes, as a basis for the subsequent discussion of how mTOR may regulate oligodendrocyte differentiation and myelin synthesis.

PI3K/Akt/mTOR signalling has been described in processes of cell differentiation in *Drosophila* as well as mammalian systems. In *Drosophila*, the dTOR pathway is activated downstream of insulin receptor signalling where it controls timing of neuronal differentiation in the eye (Bateman and McNeill, 2004). In mammalian systems, a role for mTOR in directing cell differentiation has been described in immune cells (for review, see Araki et al., 2011), myoblasts (Coolican et al., 1997; Sarbassov and Peterson, 1998; Cho et al., 2004) and adipocytes (Cho et al., 2004).

**Figure 3:**
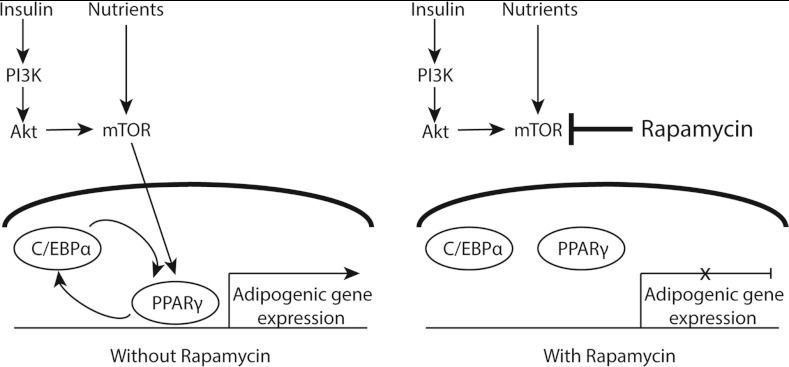
Interaction of mTOR with PPARγ and CEBP/α to regulate adipocyte differentiation In adipocytes, insulin and nutrient signalling converge on mTOR, which activates a positive-feedback loop between PPARγ and CEBP/α to regulate adipocyte gene expression. In the presence of rapamycin (an mTOR inhibitor), inactivation of PPARγ prevents adipocyte gene transcription. Figures adapted from Kim and Chen (2004).

Adipocyte differentiation is perhaps one of the best characterized and most relevant differentiation pathways from extracellular signalling to mTOR to gene transcription (Cho et al., 2004). Adipocyte differentiation involves synthesizing and storing triglycerols and mobilizing them as needed. This is a complex system, regulated by at least three transcription factors: C/EBP (CCAAT/enhancer-binding protein), PPARγ (peroxisome-proliferator-activated-receptor γ) and SREBP-1 (sterol-regulatory-element-binding protein; also called adipocyte determination and differentiation-dependent factor 1, discussed below). PPARγ is a member of a superfamily of nuclear receptors that is a critical activator of genes regulating cholesterol, fatty acids, triglycerols and phospholipid synthesis. Adipocyte differentiation is stimulated by insulin, which binds to its receptors on the cell surface, inducing phosphorylation of IRS (insulin receptor substrate) proteins. IRS then elicits the conversion of lipid messengers at the plasma membrane to recruit and activate PI3K and Akt. The transcription factors regulating adipocyte differentiation are subsequently activated in an mTORC1-dependent manner. Rapamycin studies show that inhibiting mTORC1 specifically disrupts the positive transcriptional feedback loop between C/EBP and PPARγ, blocking the transactivation activity of PPAR-γ on its target genes (Kim and Chen, 2004) (Figure 3).

#### Lipid biosynthesis and TOR

The role of mTOR in lipid biosynthesis described above in adipocytes occurs in other cells such as hepatocytes, and may well occur in oligodendrocytes. Akt-dependent lipogenesis is mediated through mTORC1, which regulates SREBP-1, a bHLH (basic helix–loop–helix) transcription factor that induces expression of genes that control fatty acid and cholesterol biosynthesis (Porstmann et al., 2008). Recent studies suggest that mTORC1 regulates SREBP-1 activity through lipin-1, a phosphatidic acid phosphatase that inhibits SREBP-1 activity by displacing it from DNA (Peterson et al., 2011). Lipin-1 is multi-phosphorylated by mTORC1 to restrict it to the cytoplasm, thereby allowing SREBP-1 to function in the nucleus. When dephosphorylated, lipin-1 moves in to the nucleus to suppress SREBP-1 transcriptional activity.

While mTORC2 has less direct impact on lipid biosynthesis, rapamycin-insensitive regulation of cholesterol biosynthesis in some cell systems suggests a role for mTORC2 (Wang et al., 2011). Conditional deletion of rictor in liver blocked the response to insulin, and reduced SREBP expression and thereby hepatic lipogenesis, which is further evidence of an mTORC2-dependent lipogenesis pathway (Yuan et al., 2012). In yeast and *Caenorhabditis elegans*, TORC2 has been identified as an important regulator of ceramide synthesis, through its downstream target SGK. Thus, TORC2 seems to play selective roles in lipid biosynthesis in some systems (for review, see Laplante and Sabatini, 2009).

This extensive literature makes it clear that both mTOR complexes impact processes of cell differentiation in several cell types, and that an important component of this in some cells includes regulating cholesterol and lipid synthetic pathways. The potential importance of this in oligodendrocyte differentiation and myelin synthesis will be discussed in more detail below.

## mTOR IN OLIGODENDROCYTE DIFFERENTIATION AND CNS/PNS MYELINATION

In the CNS, a series of studies in the past three years has demonstrated the importance of the Akt/mTOR pathway in oligodendrocyte development. Both *in vivo* and *in vitro* studies demonstrate effectively that mTOR is important in CNS myelination. Transgenic mice overexpressing constitutively active Akt in oligodendrocytes show increased expression of mTOR and increased myelination (Flores et al., 2008). Long-term exposure to rapamycin prevents the hypermyelinating phenotype in these mice as well as normal developmental myelination, but has no effect on myelin maintenance in the normal adult CNS (Narayanan et al., 2009). *In vitro* work similarly utilized rapamycin to inhibit mTOR throughout the oligodendrocyte lineage (Tyler et al., 2009; Tyler et al., 2011). These studies confirmed the importance of mTOR in myelination and provided the additional insight that mTOR regulates oligodendrocyte differentiation. A more recent *in vitro* study supports mTOR function in oligodendrocyte differentiation (Guardiola-Diaz et al., 2012), although the timing of mTOR effects appear to vary depending on *in vitro* conditions (Tyler et al., 2009; Guardiola-Diaz et al., 2012). Taken together, *in vitro* and *in vivo* works identified mTOR as a critical signalling kinase in both oligodendrocyte lineage differentiation and myelination.

The initial studies on mTOR in developing oligodendroglia support roles for both mTORC1 and mTORC2 (Tyler et al., 2009). Both complexes form, and downstream targets of mTORC1 (p70S6K and 4EBP) and mTORC2 (Akt473) are phosphorylated during oligodendrocyte differentiation *in vitro* (Tyler et al., 2009). Interestingly, however, whereas siRNA (small interfering RNA) knockdown of either raptor or rictor inhibits MBP (myelin basic protein) protein expression, only knockdown of rictor (mTORC2) reduces myelin protein mRNA expression (Tyler et al., 2009).

The initial studies on Akt and mTOR in oligodendroglia were followed by the recent demonstration that the conditional knockout of mTOR in Schwann cells has a significant impact on Schwann cell myelination (Sherman et al., 2012). While radial sorting of myelinated PNS axons occurs, as does the initial wrapping of axons by mTOR-deficient Schwann cells, the extensive increase in myelin that occurs during postnatal growth is greatly decreased, and axon diameters are grossly reduced. Very little increase in myelin thickness is observed beyond that seen at very early ages.

### Upstream of mTOR in the PNS and CNS

In both oligodendrocytes and Schwann cells, the conditional knockout of PTEN (phosphatase and tensin homologue deleted on chromosome 10), the phosphatase inhibiting PIP3 and the upstream inhibitor of PI3K/Akt, induces a hypermyelinating phenotype. This is likely mediated by mTOR, since phosphorylation of p70S6K and S6 ribosomal protein downstream of mTORC1 is increased (Goebbels et al., 2010; Harrington et al., 2010). However, remyelination in the adult is not apparently regulated by PTEN, since no hypermyelination is seen after demyelination and remyelination in the adult (Harrington et al., 2010).

More recently, PTEN was identified as the signalling pathway regulating the termination of active myelination in the PNS. PTEN is initially ubiquitinated and degraded as Schwann cells begin to myelinate. Mammalian DLG1 (disc large homologue 1) is an adaptor protein that increases during active myelination to eventually stabilize PTEN, preventing its degradation. This then reduces Akt/mTOR signalling to shift the active process of myelination to a myelin maintenance state (Cotter et al., 2010).

As discussed previously, the pathway from PI3K/Akt activation to mTOR activation includes inhibition of TSC1/2, which, when active, phosphorylates and inactivates the small GTPase Rheb, the activator of mTORC1 (see Figure 1). Conditional deletion of Rheb in embryonic neural progenitors in Nestin-Cre/Rheb^fl/fl^ mice results in postnatal hypomyelination due to defects in oligodendrocyte differentiation with no discernable effects on neuronal development (Zou et al., 2011). Loss of Rheb in the neural progenitors reduces mTORC1 signalling and increases mTORC2 signalling (Zou et al., 2011) suggesting the hypomyelination is due to loss of mTORC1. Precisely where in the developmental pathway Rheb is required is not yet clear, since nestin-Cre-driven recombination results in deletion of Rheb early in the lineage.

In contrast to the conditional Rheb deletion, genetic deletion of TSC1 in embryonic neural progenitor cells in mice results in neuronal dysfunction, epilepsy and premature death (Goto et al., 2011). These mice also are hypomyelinated, similar to reports of hypomyelination in humans with TSC mutations (Makki et al., 2007; Arulrajah et al., 2009). These reports are counter-intuitive, since the known pathway suggests that deletion of TSC function within the oligodendrocyte lineage should lead to hyperactivation of mTOR signalling. Two possible explanations for the phenoytpe with TSC1 deletion in neural progenitors are (i) these are not oligodendroglial-selective TSC1 deletions, suggesting that signals from other TSC1-deleted cells may impair myelination; and (ii) recent reports suggest that whereas TSC1/TSC2 inhibits mTORC1 via inhibiting Rheb, the TSC complex directly activates mTORC2 (Yang et al., 2006b; Huang et al., 2008; Zou et al., 2011). Thus, TSC loss could have a negative effect selectively on mTORC2 and thereby oligodendrocyte development.

### Extracellular mediators of PI3K/Akt/mTOR signalling in oligodendrocytes

Extracellular factors with demonstrated roles in promoting oligodendrocyte differentiation and/or CNS myelination through stimulation of the PI3K/Akt pathway include IGF-I, neuregulins and thyroid hormone. These factors are thus likely and, in some cases, known regulators of mTOR during oligodendrocyte development. Both IGF-I and neuregulin are potent stimulators of the PI3K/Akt pathway. However, they, along with thyroid hormone, were initially identified as important regulators of OPC and oligodendrocyte survival *in vitro*. Thus, a unique role for signalling from these molecules via PI3K/Akt specifically to regulate oligodendrocyte differentiation and myelination processes has been difficult to elucidate, although recent studies provide some insight.

#### Insulin-like growth factors

The functions of IGF-I in oligodendrocyte development *in vivo* were revealed initially from experiments showing that overexpression of IGF-I in transgenic mice results in increased brain growth and myelination (Carson et al., 1993; Ye et al., 1995). Subsequent gene knock-out studies further demonstrated that deletion of IGF-I results in reduced brain size and hypomyelination (Beck et al., 1995; Ye et al., 2002). These effects were originally attributed to IGF-I stimulation of myelin synthesis, supporting previous *in vitro* data showing that IGF-I promotes production of myelin-specific genes in differentiating oligodendrocytes (McMorris and McKinnon, 1996). However, cells at all stages of the oligodendrocyte lineage express the IGF-IR (IGF type 1 receptor) (McMorris et al., 1986; McMorris et al., 1993; McMorris and McKinnon, 1996), the major signalling receptor for IGF-I and -II. Studies using conditional deletion of IGF-1R in the oligodendrocyte lineage support functions for IGF signalling at multiple stages in the lineage including OPC proliferation and survival as well as myelin synthesis (Zeger et al., 2007).

Studies on the role of the IGF-IR in oligodendrocytes have been complicated by the fact that this receptor is also activated by insulin in the micromolar range (LeRoith et al., 1995), concentrations commonly used in chemically defined culture media for primary cells including embryonic neurons, OPCs and oligodendrocytes (Bottenstein et al., 1980; McCarthy and de Vellis, 1980). However, selective IGF-I stimulation of the IGF-1R *in vitro* under conditions where insulin levels are sufficient only to stimulate the insulin receptor but not the IGF-IR revealed roles for IGF signalling *per se* in OPC proliferation and protein synthesis as well as survival (Jiang et al., 2001; Ness et al., 2002; Ness and Wood, 2002; Frederick and Wood, 2004; Cui and Almazan, 2007; Frederick et al., 2007; Romanelli et al., 2007, 2009; Bibollet-Bahena and Almazan, 2009; Min et al., 2012). The effect of IGF-I in multiple processes during development of this lineage has made it difficult to determine whether IGF-I also directly regulates oligodendrocyte differentiation. IGF-I signalling through the IGF-IR is a potent activator of PI3K/Akt and mTOR in oligodendroglia (Ness et al., 2002; Ness and Wood, 2002; Romanelli et al., 2007; Bibollet-Bahena and Almazan, 2009; Min et al., 2012). IGF-I has the unique ability to sustain Akt signalling in differentiating oligodendrocyte progenitors (Ness and Wood, 2002), supporting the hypothesis that IGF signalling upstream of PI3K/Akt/mTOR may be important for their differentiation and maturation.

#### Neuregulins

Another signalling system regulating myelination is the neuregulin/ErbB receptor system. The role of neuregulin/ErbB receptor signalling has been more effectively investigated in the PNS, where it is a major regulator of Schwann cell development at several stages including differentiation and myelination (for reviews, see Nave and Salzer, 2006; Newbern and Birchmeier, 2010). Neuregulin signalling through PI3K regulates Schwann cell differentiation (Maurel and Salzer, 2000). The amount of neuregulin type III on the surface of axons regulates the amount of myelin generated by Schwann cells *in vivo* (Michailov et al., 2004), and neuregulin/ErbB signalling also regulates the cessation of active myelination in Schwann cells (Cotter et al., 2010). One signalling pathway has been identified connecting neuregulin/ErbB receptor signalling in the PNS with myelin gene expression and myelination *per se*. The pathway involves neuregulin/ErbB activation of Akt and increased cytoplasmic calcium. Increased calcium activates calcineurin leading to the translocation of NFAT into the nucleus, where it interacts with Sox10 to bind and activate the *Egr2* (*Krox20*) gene (Kao et al., 2009), the main transcriptional regulator of myelin gene expression in Schwann cells.

Whereas the connection from neuregulin to mTOR through PI3K/Akt in myelin-producing cells is clear in the PNS, this pathway is less clear in the CNS. Neuregulins released from or present on the surface of neurons are known to play a major role in the maintenance and survival of oligodendrocytes and their progenitors *in vitro* (Canoll et al., 1996; Vartanian et al., 1997; Fernandez et al., 2000; Flores et al., 2000). Neuregulins have additional effects in the oligodendrocyte lineage, including inducing proliferation or blocking/inducing differentiation (Lemke, 1996; Burden and Yarden, 1997). They signal through both the PI3K and the MAPK pathways (Canoll et al., 1999), but the precise signalling pathways through which neuregulins induce different responses in oligodendrocyte lineage cells are still under investigation. Furthermore, in contrast to the PNS, altered neuregulin signalling in the CNS *in vivo* does not have a consistent outcome. Some papers suggest an *in vivo* impact of neuregulin on CNS myelination (Taveggia et al., 2008), but an extensive study of reduced neuregulin 1 or ErbB gene dosage in CNS neurons or oligodendrocytes, respectively, revealed normal myelination, although increased neuregulin did enhance myelination in this study (Brinkmann et al., 2008). Thus, in the CNS, it is likely that neuregulin signalling is one of several upstream factors, which activate mTOR signalling in oligodendroglia to promote myelination.

#### Thyroid hormone

Thyroid hormone has been known to regulate oligodendrocyte differentiation and myelination since the 1960s, when neonatal thyroidectomy was shown to reduce myelination and thyroid hormone replacement reversed the deficit (Balazs et al., 1969). Thyroid hormone impacts multiple stages of oligodendrocyte development, including commitment to the lineage and OPC proliferation (Barres et al., 1994; Ahlgren et al., 1997; Rodriguez-Pena, 1999). These events appear to be regulated by expression of thyroid hormone receptor α (Fauquier et al., 2011; Picou et al., 2012). Thyroid hormone also mediates oligodendrocyte differentiation in cultured oligodendrocytes and myelinating cultures (Bhat et al., 1979; Bhat et al., 1981; Almazan et al., 1985), via expression of IGF-IR (Sarlieve et al., 2004). Thyroid receptors can act directly as transcription factors, either as homodimers or as heterodimers with retinoid X receptors or other related receptors. In fact, retinoic acid acting through the related retinoic acid receptor can substitute for thyroid hormone to enhance oligodendrocyte differentiation *in vitro* (Barres et al., 1994). In the absence of ligand, thyroid receptors are transcriptional repressors, but in the presence of thyroid hormone, in myelinating oligodendrocytes, thyroid receptors can directly activate MBP and PLP (proteolipid protein) promoters (Farsetti et al., 1991; Farsetti et al., 1992; Bogazzi et al., 1994). Intriguingly, they also have nongenomic effects by direct interaction with the p85a subunit of PI3K, resulting in activation of Akt and mTOR. In fact TRβ1, the thyroid receptor mediating oligodendrocyte differentiation, directly activates Akt in pancreatic β-cells (Verga Falzacappa et al., 2007). Whether thyroid hormone impacts Akt and mTOR signalling in differentiating oligodendrocytes is unknown.

## POTENTIAL mTOR FUNCTIONS IN OLIGODENDROCYTE DIFFERENTIATION AND MYELINATION

**Figure 4:**
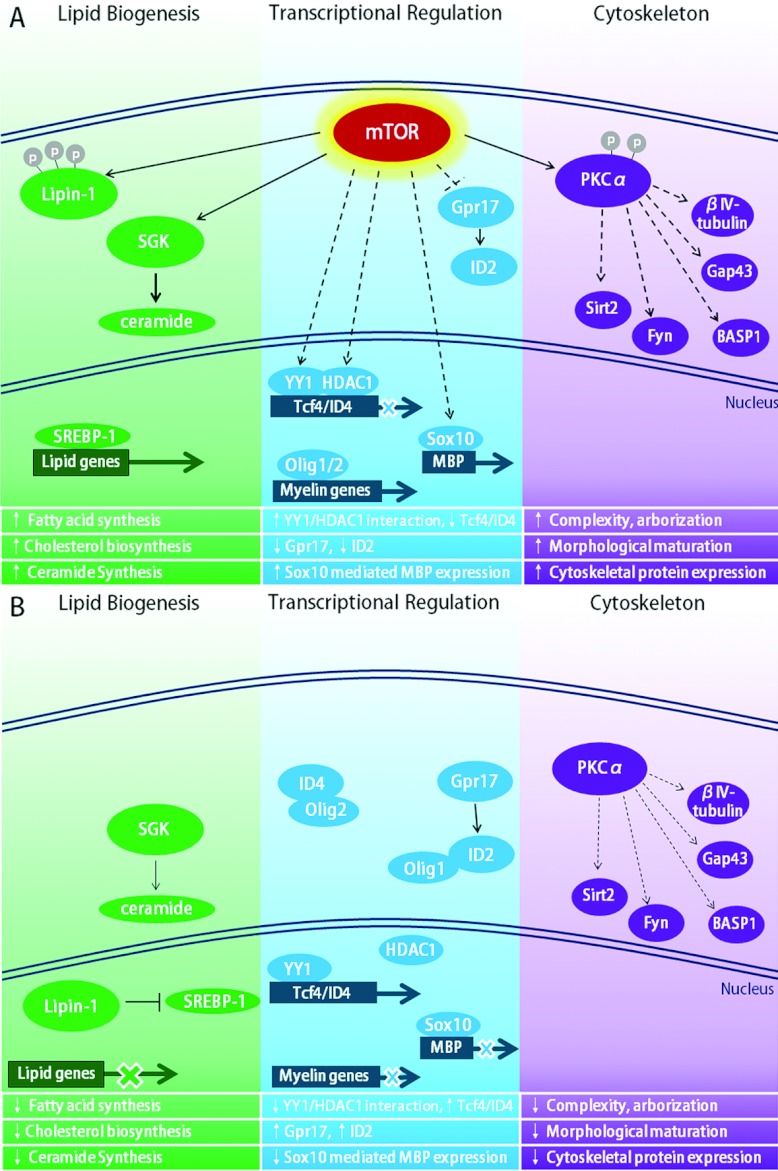
Proposed model of mTOR signalling in oligodendrocyte differentiation *Lipid biogenesis:* mTOR signalling normally promotes SGK activity, increasing ceramide synthesis, and phosphorylates Lipin-1, restricting it to the cytoplasm (**A**). In the absence of mTOR signalling (**B**), Lipin-1 remains unphosphorylated and, as such, can translocate to the nucleus where it inhibits SREBP-1 from promoting the transcription of genes involved in cholesterol and fatty acid synthesis. Thus, without mTOR signalling, lipid biogenesis is decreased. *Transcriptional regulation:* in the presence of active mTOR signalling (**A**), there is a reduction in the negative regulators of oligodendrocyte differentiation, allowing the positive regulators, such as Olig1/2 and Sox10, to promote myelin gene transcription. mTOR may function to inhibit these negative regulators through mechanisms that promote YY1 interaction with HDAC, shown to inhibit the transcription of ID4 and Tcf4, and through inhibition of Gpr17 which induces ID2 expression and localization in the nucleus. When mTOR signalling is inhibited (**B**), the negative regulators are present and active, sequestering positive regulators outside of the nucleus resulting in lower myelin gene transcription. *Cytoskeleton:* several proteins implicated in the cytoskeleton have been identified as part of the mTOR proteome. These targets may be downstream of PKC-α, the activity of which is increased upon mTOR phosphorylation (**A**). Without mTOR signalling (**B**), PKCα activity is reduced as is the expression of these cytoskeletal proteins. Dashed lines indicate hypothetical relationships based on articles cited within this review. Line thickness denotes the magnitude of the effect with thicker lines indicating a larger effect.

It is clear that the mTOR pathway is an essential mediator of PI3K/Akt signalling during oligodendrocyte development, and we can connect this pathway to several external growth factors or hormones with known roles in oligodendroglia differentiation and myelination. However, the specific targets of mTOR remain to be defined in oligodendrocytes. Identifying targets of mTOR signalling in other cell types is equally an area of current investigation by a number of laboratories. In the following sections, we discuss potential targets we predict are mTOR regulated in differentiating oligodendrocytes based on our own data as well as data obtained in studies on cell types other than oligodendrocytes. We propose several aspects of the differentiation and myelination programs for which mTOR signalling may be critical. These include nuclear regulation of differentiation, cytoskeletal rearrangements involved in morphological maturation, and processes necessary for myelin synthesis (summarized in Figure 4).

### mTOR in OPC differentiation

The initial stages of oligodendrocyte differentiation involve chromatin remodeling and transcriptional regulation. The current model for differentiation supports a co-ordinated process whereby inhibitors of differentiation are down-regulated prior to up-regulation of factors necessary for myelin gene transcription (Li et al., 2009). Several negative regulators of oligodendrocyte differentiation have been identified including the ID (inhibitor of DNA binding/differentiation) helix–loop–helix members ID2 and ID4 and TCF4/TCF7L2 (T cell factor 4). Some of these factors were initially identified as downstream targets of signalling pathways that inhibit oligodendrogenesis such as BMPs (bone morphogenetic proteins) and Wnts.

Initial studies on rapamycin inhibition of oligodendrocyte differentiation *in vitro* demonstrated elevated levels of ID2, ID4 and TCF4/TCF7L2 RNA expression, which correlate with a block in differentiation to the GalC (galactosyl cerebroside)-positive immature oligodendrocyte stage of the lineage (Tyler et al., 2009). Based on these data, we propose a role for mTOR signalling in transcriptional regulation of oligodendrocyte differentiation, specifically in down-regulating negative transcriptional regulators. Although additional transcription regulators have been identified that inhibit stages of oligodendrocyte differentiation and myelination, we focus here on the molecules and associated pathways mentioned above for which there is evidence supporting a potential role for mTOR in their regulation.

#### BMP signalling and inhibitor of DNA-binding (ID) proteins

BMPs play an important role in the regulation of oligodendrocyte development and differentiation. Cells at all stages of the oligodendrocyte lineage express BMP receptors (Mabie et al., 1997; Cheng et al., 2007). BMPs inhibit various stages of oligodendrocyte development including oligodendroglial specification, OPC differentiation into immature oligodendrocytes and the acquisition of mature myelin markers by immature oligodendrocytes. For purposes of this review, we restrict our discussion to BMP inhibition of oligodendrocyte differentiation. BMPs inhibit the differentiation of neonatal or adult OPCs to GalC-positive immature oligodendrocytes *in vitro* (Mabie et al., 1997; Grinspan et al., 2000; Cheng et al., 2007) and decrease the number of MBP-positive cells in cultures of immature oligodendrocytes undergoing differentiation (See et al., 2004; Cheng et al., 2007). Similarly, transgenic overexpression of BMP *in vivo* results in a decrease in mature oligodendrocytes throughout the brain, an effect that is inhibited *in vitro* by treating differentiating cultures from these transgenic mice with the BMP inhibitor, Noggin (Gomes et al., 2003).

Extracellular BMPs exert their effects, at least in part, through intracellular transcription factors (for a recent review, see Chen et al., 2012). BMP treatment increases mRNA and protein expression of ID2 and ID4, which inhibit oligodendrocyte development (Samanta and Kessler, 2004; Cheng et al., 2007). ID2 and ID4 proteins are inhibitory HLH transcription regulators, which lack a DNA-binding domain, and act by sequestering other HLH members. Overexpression of ID2 *in vitro* enhances PDGF-stimulated OPC proliferation and inhibits oligodendrocyte differentiation, while loss of ID2 decreases OPC proliferation and increases differentiation in culture (Wang et al., 2001). More recently, Marin-Husstege and colleagues found that overexpression of ID4 in OPCs blocks differentiation and expression of myelin genes (Marin-Husstege et al., 2006). ID4, in particular, inhibits OPC differentiation through direct binding to Olig1 and Olig2, class B HLH transcription factors that promote specification and differentiation of oligodendrocytes (Lu et al., 2002; Mekki-Dauriac et al., 2002; Rowitch et al., 2002; Takebayashi et al., 2002; Zhou and Anderson, 2002; Rowitch, 2004; Ligon et al., 2006). BMPs are thought to regulate the subcellular localization of Olig1 and Olig2 through induction of ID4, which directly binds to Olig1/2 and sequesters them from the nucleus (Samanta and Kessler, 2004). The demonstration that mTOR inhibition results in elevated levels of ID2 and ID4 and prevents OPC differentiation (Tyler et al., 2009) is suggestive of a role for mTOR either directly, by decreasing ID2/ID4 expression, or indirectly, by suppressing BMP signalling.

#### Wnt signalling and TCF7L2/TCF4

In the developing nervous system, Wnt is expressed in the dorsal cervical spinal cord (Shimizu et al., 2005). Because the formation of O4-positive OPCs is inhibited in the dorsal spinal cord, it has been postulated that dorsal factors such as Wnt negatively regulate oligodendrocyte development. In support of this concept, OPCs treated with Wnt3a ligand retain their bipolar morphology, failing to differentiate into immature oligodendrocytes (Shimizu et al., 2005; Feigenson et al., 2009; Feigenson et al., 2011). The effect of Wnt on oligodendrocyte differentiation is mediated through the canonical Wnt pathway, since it can be replicated with constitutively active β-catenin or in OPCs from mice lacking Axin2, an inhibitor of the canonical Wnt pathway (Feigenson et al., 2009; Ye et al., 2009; Fancy et al., 2011). Stabilization or addition of Wnt inhibitors prevents this block in differentiation (Shimizu et al., 2005; Feigenson et al., 2009; Fancy et al., 2011; Feigenson et al., 2011). *In vivo*, mice expressing constitutively active β-catenin or lacking Axin2 have fewer PLP-positive cells throughout the brain during both development and remyelination (Fancy et al., 2009; Feigenson et al., 2009; Ye et al., 2009; Fancy et al., 2011).

Interestingly, Wnt signalling may be dependent on BMP signalling to negatively regulate oligodendrocyte differentiation. Neural precursor cells that are transfected with active β-catenin and consequently fail to express PLP have increased BMP expression and increased ID2 and ID4 promoter activity (Ye et al., 2009; Feigenson et al., 2011). Moreover, PLP expression is rescued upon treatment of these cells with Noggin (Kasai et al., 2005). Similarly, OPCs that lack BMP receptors or are treated with Noggin differentiate normally when exposed to Wnt3a, suggesting BMP signalling is necessary for Wnt signalling to inhibit oligodendrocyte differentiation (Feigenson et al., 2011).

TCF7L2/TCF4 is downstream of Wnt/β-catenin canonical signalling and is specifically expressed in the oligodendrocyte lineage in the CNS (Fancy et al., 2009; Ye et al., 2009). In oligodendrocytes, TCF7L2/TCF4 inhibits MBP and PLP expression (Fu et al., 2009). TCF7L2/TCF4-null mice die at birth, but analysis of spinal cords revealed normal numbers of PDGFRα-positive OPCs that are likely unable to differentiate further, due to a lack of myelin synthesis (Ye et al., 2009). As mentioned previously, mTOR inhibition results in increased levels of TCF7L2/TCF4, suggesting a possible interaction between the mTOR and Wnt signalling pathways (Tyler et al., 2009).

#### YY1 and GPR17 (G-protein-coupled receptor 17): potential mechanisms for mTOR regulation of ID2, ID4 and TCF4/TCF7L2

Although interactions of the mTOR pathway with BMP and Wnt signalling could occur through distinct mechanisms, there is at least one transcriptional regulator, YY1, which co-regulates target genes in both pathways. YY1 is a zinc finger protein that can bind DNA, and is important for cell proliferation and differentiation (Donohoe et al., 1999). It can interact with HATs (histone acetyl transferases) and HDACs (histone deacetylases) to directly activate or repress gene promoters. Downstream of neuregulin signalling in the PNS, YY1 acts as an activator of Egr2/Krox20, the zinc finger transcription factor that regulates Schwann cell myelination (He et al., 2010). In the oligodendrocyte lineage, YY1 represses both *id4* and *Tcf7l2/Tcf4* gene promoters when in complex with HDAC1 (He et al., 2007; He and Casaccia-Bonnefil, 2008). Conditional ablation of YY1 in oligodendrocytes results in reduced OPC differentiation and myelination (He et al., 2007). Recent studies in the Oli-Neu glial cell line suggests that YY1 may also function later in the oligodendrocyte differentiation programme to inhibit PLP gene expression at the immature OL stage (Zolova and Wight, 2011).

YY1 represses the *ID4* and *Tcf7L2/Tcf4* gene promoters in oligodendrocytes (He et al., 2007; He and Casaccia-Bonnefil, 2008; Hu et al., 2008), as does mTOR activity, since rapamycin inhibition of mTOR in oligodendrocytes increases the expression of ID4 as well as TCF7L2/TCF4 (Tyler et al., 2009). Thus, it is possible that mTOR inhibition of ID4 and TCF7L2/TCF4 is through direct positive regulation of YY1. Alternatively, mTOR may enhance the interaction between YY1 and HDAC1, which is necessary for the inhibitory effects of YY1 on ID4 and TCF7L2/TCF4 transcription during oligodendrocyte differentiation. This may occur though interaction with raptor, which is the mechanism for mTORC1 regulation of YY1 and its function on mitochondrial gene promoters in other cell types (Cunningham et al., 2007).

Another mechanism for regulating ID2/4 is the GPR17 (Chen et al., 2009). GRP17 regulates ID2 expression levels and directly binds to both ID2 and ID4 proteins and promotes their translocation to the nucleus, thereby promoting their activity (Chen et al., 2009). GPR17 is a G-protein coupled receptor that is a repressor of oligodendrocyte differentiation and is expressed primarily in pre-myelinating oligodendrocytes (Chen et al., 2009; Ceruti et al., 2011). Its expression normally decreases as differentiation progresses (Chen et al., 2009). Removal or overexpression of GPR17 during myelination *in vivo* results in hyper- or hypo-myelination respectively (Chen et al., 2009). Of particular relevance is that GPR17 expression increased in a proteomic analysis of rapamycin-treated OPCs (Tyler et al., 2011). Thus, it is possible that the increase in ID2 observed in mTOR-inhibited cultures *in vitro* results from increased GPR17 protein expression (Tyler et al., 2009; Tyler et al., 2011).

### mTOR and myelin gene expression

Whether mTOR signalling also has a role in regulating expression of positive transcription regulators is not yet known. Whereas rapamycin inhibition of oligodendrocyte differentiation *in vitro* is associated with induction of ID2/4 and TCF7L2/TCF4 mRNA expression, rapamycin has no effect on mRNA expression of Olig1/2 or Nkx2.2 (Tyler et al., 2009). However, it is potentially of interest that siRNA knockdown of raptor or rictor results in different outcomes for myelin protein mRNA expression: siRNA to either raptor or rictor decreases MBP at the protein level but only knockdown of rictor decreases MBP mRNA as well as mRNAs for other myelin proteins (Tyler et al., 2009). These data suggest the possibility that mTORC2 may have a complex-specific role in regulating the factors responsible for inducing myelin protein gene expression.

### Additional mTOR targets in oligodendrocyte differentiation

#### Cytoskeletal regulation downstream of mTOR

Proteomic studies (Tyler et al., 2011) and a recent report by the Bansal laboratory (Guardiola-Diaz et al., 2012) support the hypothesis that mTOR signalling is required for cytoskeletal regulation and morphological maturation. Proteins identified and confirmed in the mTOR proteome with known roles in cytoskeleton include Fyn tyrosine kinase, Sirt2, bIV-tubulin, Gap43 and BASP-1 (Tyler et al., 2011). Fyn regulates the cytoskeleton in differentiating oligodendrocytes (Liang et al., 2004). A major mechanism for mTOR regulation of cytoskeleton in developing oligodendrocytes is likely via mTORC2. As discussed previously, mTORC2 regulates the cytoskeleton in other cell types via phosphorylation of PKCα (Facchinetti et al., 2008; Ikenoue et al., 2008).

#### mTOR regulation of myelin lipids

The prior proteomic analysis supports the hypothesis that, in addition to its impact on myelin protein gene expression, mTOR induces expression of multiple lipogenesis proteins as oligodendrocytes differentiate (Tyler et al., 2011). Fasn (fatty acid synthase) is expressed in oligodendrocytes late in brain development and is dramatically decreased in mTOR-inhibited oligodendrocytes (Saito et al., 2009; Tyler et al., 2011). Similarly, fdft1 (farnesyl-diphosphate farnesyltransferase 1), a cholesterol synthetic enzyme essential for myelin production (Saher et al., 2005), is also part of the mTOR-regulated proteome (Tyler et al., 2011). Several other lipid biosynthetic proteins including the cholesterol biosynthesis proteins Idi1 (isopentyenyl-diphosphate d-isomerase), Fdps (farnesyl pyrophosphate synthetase) and Hmgcs1 (hydroxymethylglutaryl-CoA synthase) and the Acsl3 and Acsl4 (fatty acid biosynthesis enzymes long-chain-fatty-acid-CoA ligase 3 and 4) (Tyler et al., 2011) were also identified in the mTOR proteome screen. Taken together, these studies support an essential role for mTOR signalling in lipogenesis in maturing oligodendrocytes initiating myelin production, similar to its role in other cell types as discussed in earlier sections of this review.

### Other signalling pathways

In addition to mTOR signalling, previous studies demonstrate that MAPK signalling also contributes to oligodendrocyte differentiation and/or myelination. Specifically, blocking p38 MAPK inhibits oligodendrocyte differentiation *in vitro* (Bhat et al., 2007; Fragoso et al., 2007; Haines et al., 2008; Chew et al., 2010), and loss of Erk2/MAPK or of both Erk1/2/MAPK in oligodendrocytes *in vivo* results in defects in developmental myelination (Fyffe-Maricich et al., 2011; Ishii et al., 2012). The Erk/MAPK pathway is essential for myelin thickness but appears dispensible for oligodendrocyte differentiation and myelin initiation during development (Ishii et al., 2012). It is entirely unclear where the MAPK signalling pathways converge with mTOR signalling (Mendoza et al., 2011). A recent study suggests the two pathways act in sequence temporally during oligodendrocyte differentiation *in vitro* (Guardiola-Diaz et al., 2012). However, the mTOR signalling literature also supports a direct action of Erk/MAPK signalling on the mTORC1 target, p70S6K (Lehman and Gomez-Cambronero, 2002). Furthermore, an extensive literature in the cancer field supports strong cross-talk between these two pathways, primarily through IRS-1, which is cross-inhibitory (for recent reviews, see Mendoza et al., 2011; De Luca et al., 2012). Whether the interaction of these pathways is positive or negative and the timing of their interactions are open areas of investigation that will have an important impact on our understanding of CNS myelination.

## CONCLUSIONS

mTOR signalling clearly has essential roles in developing oligodendrocytes and in myelination in the CNS and PNS. Our focus here has been to integrate the rapidly emerging data on mTOR in oligodendrocytes with literature defining mTOR function in other cell types. We have developed a working model placing mTOR downstream of known activators of PI3K/Akt signalling and upstream of a number of targets important for regulating many aspects of oligodendrocyte differentiation including nuclear transcriptional regulators, mediators of cytoskeletal organization and enzymes necessary for lipogenesis (Figure 4). As noted above, other signalling pathways are also clearly involved in myelination. How these pathways integrate to enhance myelination is an active and exciting research area. There will continue to be rapid progress in unraveling how extracellular regulators and intracellular signalling pathways co-ordinate to regulate the sequence of transcriptional and cellular events essential for the commitment, morphological and maturational changes necessary for normal myelination. These studies will also impact our understanding of abnormal myelination and processes involved in remyelination.

## References

[B1] Ahlgren SC, Wallace H, Bishop J, Neophytou C, Raff MC (1997). Effects of thyroid hormone on embryonic oligodendrocyte precursor cell development *in vivo* and *in vitro*. Mol Cell Neurosci.

[B2] Almazan G, Honegger P, Matthieu JM (1985). Triiodothyronine stimulation of oligodendroglial differentiation and myelination. A developmental study. Dev Neurosci.

[B3] Araki K, Ellebedy AH, Ahmed R (2011). TOR in the immune system. Curr Opin Cell Biol.

[B4] Arulrajah S, Ertan G, Jordan L, Tekes A, Khaykin E, Izbudak I, Huisman TA (2009). Magnetic resonance imaging and diffusion-weighted imaging of normal-appearing white matter in children and young adults with tuberous sclerosis complex. Neuroradiology.

[B5] Avruch J, Hara K, Lin Y, Liu M, Long X, Ortiz-Vega S, Yonezawa K (2006). Insulin and amino-acid regulation of mTOR signaling and kinase activity through the Rheb GTPase. Oncogene.

[B6] Balazs R, Brooksbank BW, Davison AN, Eayrs JT, Wilson DA (1969). The effect of neonatal thyroidectomy on myelination in the rat brain. Brain Res.

[B7] Baracho GV, Miletic AV, Omori SA, Cato MH, Rickert RC (2011). Emergence of the PI3-kinase pathway as a central modulator of normal and aberrant B cell differentiation. Curr Opin Immunol.

[B8] Baron W, Colognato H, ffrench-Constant C (2005). Integrin-growth factor interactions as regulators of oligodendroglial development and function. Glia.

[B9] Baron W, Decker L, Colognato H, ffrench-Constant C (2003). Regulation of integrin growth factor interactions in oligodendrocytes by lipid raft microdomains. Curr Biol.

[B10] Barres BA, Lazar MA, Raff MC (1994). A novel role for thyroid hormone, glucocorticoids and retinoic acid in timing oligodendrocyte development. Development.

[B11] Bateman JM, McNeill H (2004). Temporal control of differentiation by the insulin receptor/tor pathway in *Drosophila*. Cell.

[B12] Beck K, Powell-Braxton L, Widmer H, Valverde J, Hefti F (1995). Igf1 gene disruption results in reduced brain size, CNS hypomyelination, and loss of hippocampal granule and striatal parvalbumin-containing neurons. Neuron.

[B13] Bhat NR, Rao GS, Pieringer RA (1981). Investigations on myelination *in vitro*. Regulation of sulfolipid synthesis by thyroid hormone in cultures of dissociated brain cells from embryonic mice. J Biol Chem.

[B14] Bhat NR, Zhang P, Mohanty SB (2007). p38 MAP kinase regulation of oligodendrocyte differentiation with CREB as a potential target. Neurochem Res.

[B15] Bhat NR, Sarlieve LL, Rao GS, Pieringer RA (1979). Investigations on myelination *in vitro*. Regulation by thyroid hormone in cultures of dissociated brain cells from embryonic mice. J Biol Chem.

[B16] Bibollet-Bahena O, Almazan G (2009). IGF-1-stimulated protein synthesis in oligodendrocyte progenitors requires PI3K/mTOR/Akt and MEK/ERK pathways. J Neurochem.

[B17] Bogazzi F, Hudson LD, Nikodem VM (1994). A novel heterodimerization partner for thyroid hormone receptor. Peroxisome proliferator-activated receptor. J Biol Chem.

[B18] Bottenstein J, Skaper S, Baron S, Sato G (1980). Selective survival of neurons from chick embryo sensory ganglionic dissociates utilizing serum-free supplemented medium. Exp Cell Res.

[B19] Brinkmann BG, Agarwal A, Sereda MW, Garratt AN, Muller T, Wende H, Stassart RM, Nawaz S, Humml C, Velanac V, Radyushkin K, Goebbels S, Fischer TM, Franklin RJ, Lai C, Ehrenreich H, Birchmeier C, Schwab MH, Nave KA (2008). Neuregulin-1/ErbB signaling serves distinct functions in myelination of the peripheral and central nervous system. Neuron.

[B20] Burden S, Yarden Y (1997). Neuregulins and their receptors: a versatile signaling module in organogenesis and oncogenesis. Neuron.

[B21] Canoll PD, Kraemer R, Teng KK, Marchionni MA, Salzer JL (1999). GGF/neuregulin induces a phenotypic reversion of oligodendrocytes. Mol Cell Neurosci.

[B22] Canoll PD, Musacchio JM, Hardy R, Reynolds R, Marchionni MA, Salzer JL (1996). GGF/neuregulin is a neuronal signal that promotes the proliferation and survival and inhibits the differentiation of oligodendrocyte progenitors. Neuron.

[B23] Carson M, Behringer R, Brinster R, McMorris F (1993). Insulin-like growth factor I increases brain growth and central nervous system myelination in transgenic mice. Neuron.

[B24] Carson RP, Fu C, Winzenburger P, Ess KC (2013). Deletion of Rictor in neural progenitor cells reveals contributions of mTORC2 signaling to tuberous sclerosis complex. Hum Mol Genet.

[B25] Ceruti S, Vigano F, Boda E, Ferrario S, Magni G, Boccazzi M, Rosa P, Buffo A, Abbracchio MP (2011). Expression of the new P2Y-like receptor GPR17 during oligodendrocyte precursor cell maturation regulates sensitivity to ATP-induced death. Glia.

[B26] Chen CH, Sarbassov DD (2011). The mTOR (mammalian target of rapamycin) kinase maintains integrity of mTOR complex 2. J Biol Chem.

[B27] Chen XS, Zhang YH, Cai QY, Yao ZX (2012). ID2: a negative transcription factor regulating oligodendroglia differentiation. J Neurosci Res.

[B28] Chen Y, Wu H, Wang S, Koito H, Li J, Ye F, Hoang J, Escobar SS, Gow A, Arnett HA, Trapp BD, Karandikar NJ, Hsieh J, Lu QR (2009). The oligodendrocyte-specific G protein-coupled receptor GPR17 is a cell-intrinsic timer of myelination. Nat Neurosci.

[B29] Cheng X, Wang Y, He Q, Qiu M, Whittemore SR, Cao Q (2007). Bone morphogenetic protein signaling and olig1/2 interact to regulate the differentiation and maturation of adult oligodendrocyte precursor cells. Stem Cells.

[B30] Chew LJ, Coley W, Cheng Y, Gallo V (2010). Mechanisms of regulation of oligodendrocyte development by p38 mitogen-activated protein kinase. J Neurosci.

[B31] Cho HJ, Park J, Lee HW, Lee YS, Kim JB (2004). Regulation of adipocyte differentiation and insulin action with rapamycin. Biochem Biophys Res Commun.

[B32] Colognato H, Tzvetanova ID (2011). Glia unglued: how signals from the extracellular matrix regulate the development of myelinating glia. Dev Neurobiol.

[B33] Coolican SA, Samuel DS, Ewton DZ, McWade FJ, Florini JR (1997). The mitogenic and myogenic actions of insulin-like growth factors utilize distinct signaling pathways. J Biol Chem.

[B34] Cotter L, Ozcelik M, Jacob C, Pereira JA, Locher V, Baumann R, Relvas JB, Suter U, Tricaud N (2010). Dlg1-PTEN interaction regulates myelin thickness to prevent damaging peripheral nerve overmyelination. Science.

[B35] Cui QL, Almazan G (2007). IGF-I-induced oligodendrocyte progenitor proliferation requires PI3K/Akt, MEK/ERK, and Src-like tyrosine kinases. J Neurochem.

[B36] Cunningham JT, Rodgers JT, Arlow DH, Vazquez F, Mootha VK, Puigserver P (2007). mTOR controls mitochondrial oxidative function through a YY1-PGC-1alpha transcriptional complex. Nature.

[B37] Dalle Pezze P, Sonntag AG, Thien A, Prentzell MT, Godel M, Fischer S, Neumann-Haefelin E, Huber TB, Baumeister R, Shanley DP, Thedieck K (2012). A dynamic network model of mTOR signaling reveals TSC-independent mTORC2 regulation. Sci Signaling.

[B38] Dann SG, Thomas G (2006). The amino acid sensitive TOR pathway from yeast to mammals. FEBS Lett.

[B39] De Luca A, Maiello MR, D’Alessio A, Pergameno M, Normanno N (2012). The RAS/RAF/MEK/ERK and the PI3K/AKT signalling pathways: role in cancer pathogenesis and implications for therapeutic approaches. Expert Opin Ther Targets.

[B40] Dibble CC, Asara JM, Manning BD (2009). Characterization of Rictor phosphorylation sites reveals direct regulation of mTOR complex 2 by S6K1. Mol Cell Biol.

[B41] Donohoe ME, Zhang X, McGinnis L, Biggers J, Li E, Shi Y (1999). Targeted disruption of mouse Yin Yang 1 transcription factor results in peri-implantation lethality. Mol Cell Biol.

[B42] Dudek H, Datta SR, Franke TF, Birnbaum MJ, Yao R, Cooper GM, Segal RA, Kaplan DR, Greenberg ME (1997). Regulation of neuronal survival by the serine–threonine protein kinase Akt. Science.

[B43] Dunlop EA, Tee AR (2009). Mammalian target of rapamycin complex 1: signalling inputs, substrates and feedback mechanisms. Cell Signal.

[B44] Duvel K, Yecies JL, Menon S, Raman P, Lipovsky AI, Souza AL, Triantafellow E, Ma Q, Gorski R, Cleaver S, Vander Heiden MG, MacKeigan JP, Finan PM, Clish CB, Murphy LO, Manning BD (2010). Activation of a metabolic gene regulatory network downstream of mTOR complex 1. Mol Cell.

[B45] Ebner S, Dunbar M, McKinnon RD (2000). Distinct roles for PI3K in proliferation and survival of oligodendrocyte progenitor cells. J Neurosci Res.

[B46] Emery B (2010a). Regulation of oligodendrocyte differentiation and myelination. Science.

[B47] Emery B (2010b). Transcriptional and post-transcriptional control of CNS myelination. Curr Opin Neurobiol.

[B48] Eyermann C, Czaplinski K, Colognato H (2012). Dystroglycan promotes filopodial formation and process branching in differentiating oligodendroglia. J Neurochem.

[B49] Facchinetti V, Ouyang W, Wei H, Soto N, Lazorchak A, Gould C, Lowry C, Newton AC, Mao Y, Miao RQ, Sessa WC, Qin J, Zhang P, Su B, Jacinto E (2008). The mammalian target of rapamycin complex 2 controls folding and stability of Akt and protein kinase C. EMBO J.

[B50] Fancy SP, Baranzini SE, Zhao C, Yuk DI, Irvine KA, Kaing S, Sanai N, Franklin RJ, Rowitch DH (2009). Dysregulation of the Wnt pathway inhibits timely myelination and remyelination in the mammalian CNS. Genes Dev.

[B51] Fancy SP, Harrington EP, Yuen TJ, Silbereis JC, Zhao C, Baranzini SE, Bruce CC, Otero JJ, Huang EJ, Nusse R, Franklin RJ, Rowitch DH (2011). Axin2 as regulatory and therapeutic target in newborn brain injury and remyelination. Nat Neurosci.

[B52] Farsetti A, Mitsuhashi T, Desvergne B, Robbins J, Nikodem VM (1991). Molecular basis of thyroid hormone regulation of myelin basic protein gene expression in rodent brain. J Biol Chem.

[B53] Farsetti A, Desvergne B, Hallenbeck P, Robbins J, Nikodem VM (1992). Characterization of myelin basic protein thyroid hormone response element and its function in the context of native and heterologous promoter. J Biol Chem.

[B54] Fauquier T, Romero E, Picou F, Chatonnet F, Nguyen XN, Quignodon L, Flamant F (2011). Severe impairment of cerebellum development in mice expressing a dominant-negative mutation inactivating thyroid hormone receptor alpha1 isoform. Dev Biol.

[B55] Feigenson K, Reid M, See J, Crenshaw EB, Grinspan JB (2009). Wnt signaling is sufficient to perturb oligodendrocyte maturation. Mol Cell Neurosci.

[B56] Feigenson K, Reid M, See J, Crenshaw EB, Grinspan JB (2011). Canonical Wnt signalling requires the BMP pathway to inhibit oligodendrocyte maturation. ASN NEURO.

[B57] Fernandez A, Yakar S, Stannard B, LeRoith D (2000). A dominant-negative insulin-like growth factor-I receptor in skeletal muscle causes early postnatal growth retardation in mice. Endocrine Society 82nd Annual Meeting.

[B58] Fishwick KJ, Li RA, Halley P, Deng P, Storey KG (2010). Initiation of neuronal differentiation requires PI3-kinase/TOR signalling in the vertebrate neural tube. Dev Biol.

[B59] Flores AI, Mallon BS, Matsui T, Ogawa W, Rosenzweig A, Okamoto T, Macklin WB (2000). Akt-mediated survival of oligodendrocytes induced by neuregulins. J Neurosci.

[B60] Flores AI, Narayanan SP, Morse EN, Shick HE, Yin X, Kidd G, Avila RL, Kirschner DA, Macklin WB (2008). Constitutively active Akt induces enhanced myelination in the CNS. J Neurosci.

[B61] Fragoso G, Haines JD, Roberston J, Pedraza L, Mushynski WE, Almazan G (2007). p38 Mitogen-activated protein kinase is required for central nervous system myelination. Glia.

[B62] Franke TF, Kaplan DR, Cantley LC (1997). PI3K: downstream AKTion blocks apoptosis. Cell.

[B63] Franke TF, Yang SI, Chan TO, Datta K, Kazlauskas A, Morrison DK, Kaplan DR, Tsichlis PN (1995). The protein kinase encoded by the Akt proto-oncogene is a target of the PDGF-activated phosphatidylinositol 3-kinase. Cell.

[B64] Frederick TJ, Wood TL (2004). IGF-I and FGF-2 coordinately enhance cyclin D1 and cyclin E-cdk2 association and activity to promote G(1) progression in oligodendrocyte progenitor cells. Mol Cell Neurosci.

[B65] Frederick TJ, Min J, Altieri SC, Mitchell NE, Wood TL (2007). Synergistic induction of cyclin D1 in oligodendrocyte progenitor cells by IGF-I and FGF-2 requires differential stimulation of multiple signaling pathways. Glia.

[B66] Fu H, Cai J, Clevers H, Fast E, Gray S, Greenberg R, Jain MK, Ma Q, Qiu M, Rowitch DH, Taylor CM, Stiles CD (2009). A genome-wide screen for spatially restricted expression patterns identifies transcription factors that regulate glial development. J Neurosci.

[B67] Fyffe-Maricich SL, Karlo JC, Landreth GE, Miller RH (2011). The ERK2 mitogen-activated protein kinase regulates the timing of oligodendrocyte differentiation. J Neurosci.

[B68] Gardner S, Anguiano M, Rotwein P (2012). Defining Akt actions in muscle differentiation. Am J Physiol Cell Physiol.

[B69] Goebbels S, Oltrogge JH, Kemper R, Heilmann I, Bormuth I, Wolfer S, Wichert SP, Mobius W, Liu X, Lappe-Siefke C, Rossner MJ, Groszer M, Suter U, Frahm J, Boretius S, Nave KA (2010). Elevated phosphatidylinositol 3,4,5-trisphosphate in glia triggers cell-autonomous membrane wrapping and myelination. J Neurosci.

[B70] Gomes WA, Mehler MF, Kessler JA (2003). Transgenic overexpression of BMP4 increases astroglial and decreases oligodendroglial lineage commitment. Dev Biol.

[B71] Goto J, Talos DM, Klein P, Qin W, Chekaluk YI, Anderl S, Malinowska IA, Di Nardo A, Bronson RT, Chan JA, Vinters HV, Kernie SG, Jensen FE, Sahin M, Kwiatkowski DJ (2011). Regulable neural progenitor-specific Tsc1 loss yields giant cells with organellar dysfunction in a model of tuberous sclerosis complex. Proc Natl Acad Sci USA.

[B72] Grinspan JB, Edell E, Carpio DF, Beesley JS, Lavy L, Pleasure D, Golden JA (2000). Stage-specific effects of bone morphogenetic proteins on the oligodendrocyte lineage. J Neurobiol.

[B73] Gu Y, Lindner J, Kumar A, Yuan W, Magnuson MA (2010). Rictor/mTORC2 is essential for maintaining a balance between beta-cell proliferation and cell size. Diabetes.

[B74] Guardiola-Diaz HM, Ishii A, Bansal R (2012). Erk1/2 MAPK and mTOR signaling sequentially regulates progression through distinct stages of oligodendrocyte differentiation. Glia.

[B75] Guertin DA, Guntur KV, Bell GW, Thoreen CC, Sabatini DM (2006a). Functional genomics identifies TOR-regulated genes that control growth and division. Curr Biol.

[B76] Guertin DA, Stevens DM, Thoreen CC, Burds AA, Kalaany NY, Moffat J, Brown M, Fitzgerald KJ, Sabatini DM (2006b). Ablation in mice of the mTORC components raptor, rictor, or mLST8 reveals that mTORC2 is required for signaling to Akt-FOXO and PKCalpha, but not S6K1. Dev Cell.

[B77] Guertin DA, Stevens DM, Saitoh M, Kinkel S, Crosby K, Sheen JH, Mullholland DJ, Magnuson MA, Wu H, Sabatini DM (2009). mTOR complex 2 is required for the development of prostate cancer induced by Pten loss in mice. Cancer Cell.

[B78] Haines JD, Fragoso G, Hossain S, Mushynski WE, Almazan G (2008). p38 Mitogen-activated protein kinase regulates myelination. J Mol Neurosci.

[B79] Hannan KM, Brandenburger Y, Jenkins A, Sharkey K, Cavanaugh A, Rothblum L, Moss T, Poortinga G, McArthur GA, Pearson RB, Hannan RD (2003). mTOR-dependent regulation of ribosomal gene transcription requires S6K1 and is mediated by phosphorylation of the carboxy-terminal activation domain of the nucleolar transcription factor UBF. Mol Cell Biol.

[B80] Hara K, Maruki Y, Long X, Yoshino K, Oshiro N, Hidayat S, Tokunaga C, Avruch J, Yonezawa K (2002). Raptor, a binding partner of target of rapamycin (TOR), mediates TOR action. Cell.

[B81] Harrington EP, Zhao C, Fancy SP, Kaing S, Franklin RJ, Rowitch DH (2010). Oligodendrocyte PTEN is required for myelin and axonal integrity, not remyelination. Ann Neurol.

[B82] He Y, Casaccia-Bonnefil P (2008). The Yin and Yang of YY1 in the nervous system. J Neurochem.

[B83] He Y, Kim JY, Dupree J, Tewari A, Melendez-Vasquez C, Svaren J, Casaccia P (2010). Yy1 as a molecular link between neuregulin and transcriptional modulation of peripheral myelination. Nat Neurosci.

[B84] He Y, Dupree J, Wang J, Sandoval J, Li J, Liu H, Shi Y, Nave KA, Casaccia-Bonnefil P (2007). The transcription factor Yin Yang 1 is essential for oligodendrocyte progenitor differentiation. Neuron.

[B85] Hoshii T, Tadokoro Y, Naka K, Ooshio T, Muraguchi T, Sugiyama N, Soga T, Araki K, Yamamura K, Hirao A (2012). mTORC1 is essential for leukemia propagation but not stem cell self-renewal. J Clin Invest.

[B86] Hu JG, Fu SL, Wang YX, Li Y, Jiang XY, Wang XF, Qiu MS, Lu PH, Xu XM (2008). Platelet-derived growth factor-AA mediates oligodendrocyte lineage differentiation through activation of extracellular signal-regulated kinase signaling pathway. Neuroscience.

[B87] Huang J, Dibble CC, Matsuzaki M, Manning BD (2008). The TSC1-TSC2 complex is required for proper activation of mTOR complex 2. Mol Cell Biol.

[B88] Hudson CC, Liu M, Chiang GG, Otterness DM, Loomis DC, Kaper F, Giaccia AJ, Abraham RT (2002). Regulation of hypoxia-inducible factor 1alpha expression and function by the mammalian target of rapamycin. Mol Cell Biol.

[B89] Huo Y, Iadevaia V, Proud CG (2011). Differing effects of rapamycin and mTOR kinase inhibitors on protein synthesis. Biochem Soc Trans.

[B90] Hwang M, Perez CA, Moretti L, Lu B (2008). The mTOR signaling network: insights from its role during embryonic development. Curr Med Chem.

[B91] Ikenoue T, Inoki K, Yang Q, Zhou X, Guan KL (2008). Essential function of TORC2 in PKC and Akt turn motif phosphorylation, maturation and signalling. EMBO J.

[B92] Ishii A, Fyffe-Maricich SL, Furusho M, Miller RH, Bansal R (2012). ERK1/ERK2 MAPK signaling is required to increase myelin thickness independent of oligodendrocyte differentiation and initiation of myelination. J Neurosci.

[B93] Jacinto E, Loewith R, Schmidt A, Lin S, Ruegg MA, Hall A, Hall MN (2004). Mammalian TOR complex 2 controls the actin cytoskeleton and is rapamycin insensitive. Nat Cell Biol.

[B94] Jiang F, Frederick TJ, Wood TL (2001). IGF-I synergizes with FGF-2 to stimulate oligodendrocyte progenitor entry into the cell cycle. Dev Biol.

[B95] Julien LA, Carriere A, Moreau J, Roux PP (2010). mTORC1-activated S6K1 phosphorylates Rictor on threonine 1135 and regulates mTORC2 signaling. Mol Cell Biol.

[B96] Kalaitzidis D, Sykes SM, Wang Z, Punt N, Tang Y, Ragu C, Sinha AU, Lane SW, Souza AL, Clish CB, Anastasiou D, Gilliland DG, Scadden DT, Guertin DA, Armstrong SA (2012). mTOR complex 1 plays critical roles in hematopoiesis and Pten-loss-evoked leukemogenesis. Cell Stem Cell.

[B97] Kantidakis T, Ramsbottom BA, Birch JL, Dowding SN, White RJ (2010). mTOR associates with TFIIIC, is found at tRNA and 5S rRNA genes, and targets their repressor Maf1. Proc Natl Acad Sci USA.

[B98] Kao SC, Wu H, Xie J, Chang CP, Ranish JA, Graef IA, Crabtree GR (2009). Calcineurin/NFAT signaling is required for neuregulin-regulated Schwann cell differentiation. Science.

[B99] Kasai M, Satoh K, Akiyama T (2005). Wnt signaling regulates the sequential onset of neurogenesis and gliogenesis via induction of BMPs. Genes Cells.

[B100] Kennedy SG, Wagner AJ, Conzen SD, Jordan J, Bellacosa A, Tsichlis PN, Hay N (1997). The PI 3-kinase/Akt signaling pathway delivers an anti-apoptotic signal. Genes Dev.

[B101] Kim DH, Sarbassov DD, Ali SM, King JE, Latek RR, Erdjument-Bromage H, Tempst P, Sabatini DM (2002). mTOR interacts with raptor to form a nutrient-sensitive complex that signals to the cell growth machinery. Cell.

[B102] Kim DH, Sarbassov DD, Ali SM, Latek RR, Guntur KV, Erdjument-Bromage H, Tempst P, Sabatini DM (2003). GbetaL, a positive regulator of the rapamycin-sensitive pathway required for the nutrient-sensitive interaction between raptor and mTOR. Mol Cell.

[B103] Kim JE, Chen J (2004). regulation of peroxisome proliferator-activated receptor-gamma activity by mammalian target of rapamycin and amino acids in adipogenesis. Diabetes.

[B104] Kovacina KS, Park GY, Bae SS, Guzzetta AW, Schaefer E, Birnbaum MJ, Roth RA (2003). Identification of a proline-rich Akt substrate as a 14-3-3 binding partner. J Biol Chem.

[B105] Kumar A, Harris TE, Keller SR, Choi KM, Magnuson MA, Lawrence JC (2008). Muscle-specific deletion of rictor impairs insulin-stimulated glucose transport and enhances Basal glycogen synthase activity. Mol Cell Biol.

[B106] Kumar A, Lawrence JC, Jung DY, Ko HJ, Keller SR, Kim JK, Magnuson MA, Harris TE (2010). Fat cell-specific ablation of rictor in mice impairs insulin-regulated fat cell and whole-body glucose and lipid metabolism. Diabetes.

[B107] Lafrenaye AD, Fuss B (2010). Focal adhesion kinase can play unique and opposing roles in regulating the morphology of differentiating oligodendrocytes. J Neurochem.

[B108] Lang CH, Frost RA, Bronson SK, Lynch CJ, Vary TC (2010). Skeletal muscle protein balance in mTOR heterozygous mice in response to inflammation and leucine. Am J Physiol Endocrinol Metab.

[B109] Laplante M, Sabatini DM (2009). An emerging role of mTOR in lipid biosynthesis. Curr Biol.

[B110] Lee K, Gudapati P, Dragovic S, Spencer C, Joyce S, Killeen N, Magnuson MA, Boothby M (2010). Mammalian target of rapamycin protein complex 2 regulates differentiation of Th1 and Th2 cell subsets via distinct signaling pathways. Immunity.

[B111] Lehman JA, Gomez-Cambronero J (2002). Molecular crosstalk between p70S6k and MAPK cell signaling pathways. Biochem Biophys Res Commun.

[B112] Lemke G (1996). Neuregulins in development. Mol Cell Neurosci.

[B113] LeRoith D, Werner H, Beitner-Johnson D, Roberts CJ (1995). Molecular and cellular aspects of the insulin-like growth factor I receptor. Endocr Rev.

[B114] Li H, He Y, Richardson WD, Casaccia P (2009). Two-tier transcriptional control of oligodendrocyte differentiation. Curr Opin Neurobiol.

[B115] Liang X, Draghi NA, Resh MD (2004). Signaling from integrins to Fyn to Rho family GTPases regulates morphologic differentiation of oligodendrocytes. J Neurosci.

[B116] Ligon KL, Fancy SP, Franklin RJ, Rowitch DH (2006). Olig gene function in CNS development and disease. Glia.

[B117] Liu L, Chen L, Chung J, Huang S (2008). Rapamycin inhibits F-actin reorganization and phosphorylation of focal adhesion proteins. Oncogene.

[B118] Liu Q, Chang JW, Wang J, Kang SA, Thoreen CC, Markhard A, Hur W, Zhang J, Sim T, Sabatini DM, Gray NS (2010). Discovery of 1-(4-(4-propionylpiperazin-1-yl)-3-(trifluoromethyl)phenyl)-9-(quinolin3-yl)benz o[h][1,6]naphthyridin-2(1H)-one as a highly potent, selective mammalian target of rapamycin (mTOR) inhibitor for the treatment of cancer. J Med Chem.

[B119] Liu Q, Kirubakaran S, Hur W, Niepel M, Westover K, Thoreen CC, Wang J, Ni J, Patricelli MP, Vogel K, Riddle S, Waller DL, Traynor R, Sanda T, Zhao Z, Kang SA, Zhao J, Look AT, Sorger PK, Sabatini DM, Gray NS (2012). Kinome-wide selectivity profiling of ATP-competitive mammalian target of rapamycin (mTOR) inhibitors and characterization of their binding kinetics. J Biol Chem.

[B120] Lu QR, Sun T, Zhu Z, Ma N, Garcia M, Stiles CD, Rowitch DH (2002). Common developmental requirement for Olig function indicates a motor neuron/oligodendrocyte connection. Cell.

[B121] Mabie PC, Mehler MF, Marmur R, Papavasiliou A, Song Q, Kessler JA (1997). Bone morphogenetic proteins induce astroglial differentiation of oligodendroglial-astroglial progenitor cells. J Neurosci.

[B122] Makki MI, Chugani DC, Janisse J, Chugani HT (2007). Characteristics of abnormal diffusivity in normal-appearing white matter investigated with diffusion tensor MR imaging in tuberous sclerosis complex. AJNR Am J Neuroradiol.

[B123] Marin-Husstege M, He Y, Li J, Kondo T, Sablitzky F, Casaccia-Bonnefil P (2006). Multiple roles of Id4 in developmental myelination: predicted outcomes and unexpected findings. Glia.

[B124] Maurel P, Salzer JL (2000). Axonal regulation of Schwann cell proliferation and survival and the initial events of myelination requires PI 3-kinase activity. J Neurosci.

[B125] McCarthy K, de Vellis J (1980). Preparation of separate astroglial and oligodendroglial cell cultures from rat cerebral tissue. J Cell Biol.

[B126] McMorris F, McKinnon R (1996). Regulation of oligodendrocyte development and CNS myelination by growth factors: Prospects for therapy of demyelinating disease. Brain Pathol.

[B127] McMorris F, Smith T, DeSalvo S, Furlanetto R (1986). Insulin-like growth factor I/somatomedin C: a potent inducer of oligodendrocyte development. Proc Natl Acad Sci USA.

[B128] McMorris F, Mozell R, Carson M, Shinar Y, Meyer R, Marchetti N (1993). Regulation of oligodendrocyte development and central nervous system myelination by insulin-like growth factors. Ann New York Acad Sci.

[B129] Mekki-Dauriac S, Agius E, Kan P, Cochard P (2002). Bone morphogenetic proteins negatively control oligodendrocyte precursor specification in the chick spinal cord. Development.

[B130] Mendoza MC, Er EE, Blenis J (2011). The Ras-ERK and PI3K-mTOR pathways: cross-talk and compensation. Trends Biochem Sci.

[B131] Michailov GV, Sereda MW, Brinkmann BG, Fischer TM, Haug B, Birchmeier C, Role L, Lai C, Schwab MH, Nave KA (2004). Axonal neuregulin-1 regulates myelin sheath thickness. Science.

[B132] Michlewski G, Sanford JR, Caceres JF (2008). The splicing factor SF2/ASF regulates translation initiation by enhancing phosphorylation of 4E-BP1. Mol Cell.

[B133] Miller RH (2002). Regulation of oligodendrocyte development in the vertebrate CNS. Prog Neurobiol.

[B134] Min J, Singh S, Fitzgerald-Bocarsly P, Wood TL (2012). Insulin-like growth factor I regulates G2/M progression through mammalian target of rapamycin signaling in oligodendrocyte progenitors. Glia.

[B135] Narayanan SP, Flores AI, Wang F, Macklin WB (2009). Akt signals through the mammalian target of rapamycin pathway to regulate CNS myelination. J Neurosci.

[B136] Nave KA, Salzer JL (2006). Axonal regulation of myelination by neuregulin 1. Curr Opin Neurobiol.

[B137] Ness JK, Wood TL (2002). Insulin-like growth factor I, but not neurotrophin-3, sustains Akt activation and provides long-term protection of immature oligodendrocytes from glutamate-mediated apoptosis. Mol Cell Neurosci.

[B138] Ness JK, Mitchell NE, Wood TL (2002). IGF-I and NT-3 signaling pathways in developing oligodendrocytes: differential regulation and activation of receptors and the downstream effector Akt. Dev Neurosci.

[B139] Ness JK, Scaduto RC, Wood TL (2004). IGF-I prevents glutamate-mediated bax translocation and cytochrome C release in O4+ oligodendrocyte progenitors. Glia.

[B140] Newbern J, Birchmeier C (2010). Nrg1/ErbB signaling networks in Schwann cell development and myelination. Semin Cell Dev Biol.

[B141] Parekh D, Ziegler W, Yonezawa K, Hara K, Parker PJ (1999). Mammalian TOR controls one of two kinase pathways acting upon nPKCdelta and nPKCepsilon. J Biol Chem.

[B142] Pearce LR, Komander D, Alessi DR (2010). The nuts and bolts of AGC protein kinases. Nat Rev Mol Cell Biol.

[B143] Pearce LR, Sommer EM, Sakamoto K, Wullschleger S, Alessi DR (2011). Protor-1 is required for efficient mTORC2-mediated activation of SGK1 in the kidney. Biochem J.

[B144] Pearce LR, Huang X, Boudeau J, Pawlowski R, Wullschleger S, Deak M, Ibrahim AF, Gourlay R, Magnuson MA, Alessi DR (2007). Identification of Protor as a novel Rictor-binding component of mTOR complex-2. Biochem J.

[B145] Peng T, Golub TR, Sabatini DM (2002). The immunosuppressant rapamycin mimics a starvation-like signal distinct from amino acid and glucose deprivation. Mol Cell Biol.

[B146] Peterson TR, Sengupta SS, Harris TE, Carmack AE, Kang SA, Balderas E, Guertin DA, Madden KL, Carpenter AE, Finck BN, Sabatini DM (2011). mTOR complex 1 regulates lipin 1 localization to control the SREBP pathway. Cell.

[B147] Pfeiffer SE, Warrington AE, Bansal R (1993). The oligodendrocyte and its many cellular processes. Trends Cell Biol.

[B148] Picou F, Fauquier T, Chatonnet F, Flamant F (2012). A bimodal influence of thyroid hormone on cerebellum oligodendrocyte differentiation. Mol Endocrinol.

[B149] Porstmann T, Santos CR, Griffiths B, Cully M, Wu M, Leevers S, Griffiths JR, Chung YL, Schulze A (2008). SREBP activity is regulated by mTORC1 and contributes to Akt-dependent cell growth. Cell Metab.

[B150] Rajasekharan S, Bin JM, Antel JP, Kennedy TE (2010). A central role for RhoA during oligodendroglial maturation in the switch from netrin-1-mediated chemorepulsion to process elaboration. J Neurochem.

[B151] Risson V, Mazelin L, Roceri M, Sanchez H, Moncollin V, Corneloup C, Richard-Bulteau H, Vignaud A, Baas D, Defour A, Freyssenet D, Tanti JF, Le-Marchand-Brustel Y, Ferrier B, Conjard-Duplany A, Romanino K, Bauché S, Hantaï D, Mueller M, Kozma SC, Thomas G, Rüegg MA, Ferry A, Pende M, Bigard X, Koulmann N, Schaeffer L, Gangloff YG (2009). Muscle inactivation of mTOR causes metabolic and dystrophin defects leading to severe myopathy. J Cell Biol.

[B152] Rodriguez-Pena A (1999). Oligodendrocyte development and thyroid hormone. J Neurobiol.

[B153] Romanelli RJ, Mahajan KR, Fulmer CG, Wood TL (2009). Insulin-like growth factor-I-stimulated Akt phosphorylation and oligodendrocyte progenitor cell survival require cholesterol-enriched membranes. J Neurosci Res.

[B154] Romanelli RJ, Lebeau AP, Fulmer CG, Lazzarino DA, Hochberg A, Wood TL (2007). IGF type-I receptor internalization and recycling mediate the sustained phosphorylation of AKT. J Biol Chem.

[B155] Rosner M, Hengstschlager M (2008). Cytoplasmic and nuclear distribution of the protein complexes mTORC1 and mTORC2: rapamycin triggers dephosphorylation and delocalization of the mTORC2 components rictor and sin1. Hum Mol Genet.

[B156] Rowitch DH (2004). Glial specification in the vertebrate neural tube. Nat Rev Neurosci.

[B157] Rowitch DH, Lu QR, Kessaris N, Richardson WD (2002). An ‘oligarchy’ rules neural development. Trends Neurosci.

[B158] Saher G, Brugger B, Lappe-Siefke C, Mobius W, Tozawa R, Wehr MC, Wieland F, Ishibashi S, Nave KA (2005). High cholesterol level is essential for myelin membrane growth. Nat Neurosci.

[B159] Saito M, Chakraborty G, Mao RF, Vadasz C (2009). Developmental profiles of lipogenic enzymes and their regulators in the neonatal mouse brain. Neurochem Res.

[B160] Samanta J, Kessler JA (2004). Interactions between ID and OLIG proteins mediate the inhibitory effects of BMP4 on oligodendroglial differentiation. Development.

[B161] Sancak Y, Thoreen CC, Peterson TR, Lindquist RA, Kang SA, Spooner E, Carr SA, Sabatini DM (2007). PRAS40 is an insulin-regulated inhibitor of the mTORC1 protein kinase. Mol Cell.

[B162] Sarbassov DD, Peterson CA (1998). Insulin receptor substrate-1 and phosphatidylinositol 3-kinase regulate extracellular signal-regulated kinase-dependent and -independent signaling pathways during myogenic differentiation. Mol Endocrinol.

[B163] Sarbassov DD, Ali SM, Sabatini DM (2005a). Growing roles for the mTOR pathway. Curr Opin Cell Biol.

[B164] Sarbassov DD, Guertin DA, Ali SM, Sabatini DM (2005b). Phosphorylation and regulation of Akt/PKB by the rictor-mTOR complex. Science.

[B165] Sarbassov DD, Ali SM, Kim DH, Guertin DA, Latek RR, Erdjument-Bromage H, Tempst P, Sabatini DM (2004). Rictor, a novel binding partner of mTOR, defines a rapamycin-insensitive and raptor-independent pathway that regulates the cytoskeleton. Curr Biol.

[B166] Sarbassov DD, Ali SM, Sengupta S, Sheen JH, Hsu PP, Bagley AF, Markhard AL, Sabatini DM (2006). Prolonged rapamycin treatment inhibits mTORC2 assembly and Akt/PKB. Mol Cell.

[B167] Sarlieve LL, Rodriguez-Pena A, Langley K (2004). Expression of thyroid hormone receptor isoforms in the oligodendrocyte lineage. Neurochem Res.

[B168] See J, Zhang X, Eraydin N, Mun SB, Mamontov P, Golden JA, Grinspan JB (2004). Oligodendrocyte maturation is inhibited by bone morphogenetic protein. Mol Cell Neurosci.

[B169] Sekulic A, Hudson CC, Homme JL, Yin P, Otterness DM, Karnitz LM, Abraham RT (2000). A direct linkage between the phosphoinositide 3-kinase-AKT signaling pathway and the mammalian target of rapamycin in mitogen-stimulated and transformed cells. Cancer Res.

[B170] Sengupta S, Peterson TR, Laplante M, Oh S, Sabatini DM (2010). mTORC1 controls fasting-induced ketogenesis and its modulation by ageing. Nature.

[B171] Sherman DL, Krols M, Wu LM, Grove M, Nave KA, Gangloff YG, Brophy PJ (2012). Arrest of myelination and reduced axon growth when Schwann cells lack mTOR. J Neurosci.

[B172] Shimizu T, Kagawa T, Wada T, Muroyama Y, Takada S, Ikenaka K (2005). Wnt signaling controls the timing of oligodendrocyte development in the spinal cord. Dev Biol.

[B173] Shor B, Wu J, Shakey Q, Toral-Barza L, Shi C, Follettie M, Yu K (2010). Requirement of the mTOR kinase for the regulation of Maf1 phosphorylation and control of RNA polymerase III-dependent transcription in cancer cells. J Biol Chem.

[B174] Siuta MA, Robertson SD, Kocalis H, Saunders C, Gresch PJ, Khatri V, Shiota C, Kennedy JP, Lindsley CW, Daws LC, Polley DB, Veenstra-Vanderweele J, Stanwood GD, Magnuson MA, Niswender KD, Galli A (2010). Dysregulation of the norepinephrine transporter sustains cortical hypodopaminergia and schizophrenia-like behaviors in neuronal rictor null mice. PLoS Biol.

[B175] Soliman GA, Acosta-Jaquez HA, Dunlop EA, Ekim B, Maj NE, Tee AR, Fingar DC (2010). mTOR Ser-2481 autophosphorylation monitors mTORC-specific catalytic activity and clarifies rapamycin mechanism of action. J Biol Chem.

[B176] Song J, Goetz BD, Baas PW, Duncan ID (2001). Cytoskeletal reorganization during the formation of oligodendrocyte processes and branches. Mol Cell Neurosci.

[B177] Sparks CA, Guertin DA (2010). Targeting mTOR: prospects for mTOR complex 2 inhibitors in cancer therapy. Oncogene.

[B178] Takebayashi H, Nabeshima Y, Yoshida S, Chisaka O, Ikenaka K (2002). The basic helix-loop-helix factor olig2 is essential for the development of motoneuron and oligodendrocyte lineages. Curr Biol.

[B179] Tang F, Wu Q, Ikenoue T, Guan KL, Liu Y, Zheng P (2012). A critical role for Rictor in T lymphopoiesis. J Immunol.

[B180] Taveggia C, Thaker P, Petrylak A, Caporaso GL, Toews A, Falls DL, Einheber S, Salzer JL (2008). Type III neuregulin-1 promotes oligodendrocyte myelination. Glia.

[B181] Thoreen CC, Kang SA, Chang JW, Liu Q, Zhang J, Gao Y, Reichling LJ, Sim T, Sabatini DM, Gray NS (2009). An ATP-competitive mammalian target of rapamycin inhibitor reveals rapamycin-resistant functions of mTORC1. J Biol Chem.

[B182] Tyler WA, Gangoli N, Gokina P, Kim HA, Covey M, Levison SW, Wood TL (2009). Activation of the mammalian target of rapamycin (mTOR) is essential for oligodendrocyte differentiation. J Neurosci.

[B183] Tyler WA, Jain MR, Cifelli SE, Li Q, Ku L, Feng Y, Li H, Wood TL (2011). Proteomic identification of novel targets regulated by the mammalian target of rapamycin pathway during oligodendrocyte differentiation. Glia.

[B184] Vander Haar E, Lee SI, Bandhakavi S, Griffin TJ, Kim DH (2007). Insulin signalling to mTOR mediated by the Akt/PKB substrate PRAS40. Nat Cell Biol.

[B185] Vartanian T, Goodearl A, Viehover A, Fischbach G (1997). Axonal neuregulin signals cells of the oligodendrocyte lineage through activation of HER4 and Schwann cells through HER2 and HER3. J Cell Biol.

[B186] Verga Falzacappa C, Petrucci E, Patriarca V, Michienzi S, Stigliano A, Brunetti E, Toscano V, Misiti S (2007). Thyroid hormone receptor TRbeta1 mediates Akt activation by T3 in pancreatic beta cells. J Mol Endocrinol.

[B187] Wang BT, Ducker GS, Barczak AJ, Barbeau R, Erle DJ, Shokat KM (2011). The mammalian target of rapamycin regulates cholesterol biosynthetic gene expression and exhibits a rapamycin-resistant transcriptional profile. Proc Natl Acad Sci USA.

[B188] Wang S, Sdrulla A, Johnson JE, Yokota Y, Barres BA (2001). A role for the helix-loop-helix protein Id2 in the control of oligodendrocyte development. Neuron.

[B189] Wegner M (2008). A matter of identity: transcriptional control in oligodendrocytes. J Mol Neurosci.

[B190] White ES, Sagana RL, Booth AJ, Yan M, Cornett AM, Bloomheart CA, Tsui JL, Wilke CA, Moore BB, Ritzenthaler JD, Roman J, Muro AF (2010). Control of fibroblast fibronectin expression and alternative splicing via the PI3K/Akt/mTOR pathway. Exp Cell Res.

[B191] Willis IM, Moir RD (2007). Integration of nutritional and stress signaling pathways by Maf1. Trends Biochem Sci.

[B192] Wullschleger S, Loewith R, Hall MN (2006). TOR signaling in growth and metabolism. Cell.

[B193] Yang Q, Inoki K, Ikenoue T, Guan KL (2006a). Identification of Sin1 as an essential TORC2 component required for complex formation and kinase activity. Genes Dev.

[B194] Yang Q, Inoki K, Kim E, Guan KL (2006b). TSC1/TSC2 and Rheb have different effects on TORC1 and TORC2 activity. Proc Natl Acad Sci USA.

[B195] Ye F, Chen Y, Hoang T, Montgomery RL, Zhao XH, Bu H, Hu T, Taketo MM, van Es JH, Clevers H, Hsieh J, Bassel-Duby R, Olson EN, Lu QR (2009). HDAC1 and HDAC2 regulate oligodendrocyte differentiation by disrupting the beta-catenin-TCF interaction. Nat Neurosci.

[B196] Ye P, Carson J, D’Ercole AJ (1995). *In vivo* actions of insulin-like growth factor-I (IGF-I) on brain myelination: studies of IGF-I and IGF binding protein (IGFBP-1) transgenic mice. J Neurosci.

[B197] Ye P, Li L, Richards G, DiAugustine RP, D’Ercole AJ (2002). Myelination is altered in insulin-like growth factor-I null mutant mice. J Neurosci.

[B198] Yuan M, Pino E, Wu L, Kacergis M, Soukas AA (2012). Identification of Akt-independent regulation of hepatic lipogenesis by mammalian target of rapamycin (mTOR) complex 2. J Biol Chem.

[B199] Zaka M, Rafi MA, Rao HZ, Luzi P, Wenger DA (2005). Insulin-like growth factor-1 provides protection against psychosine-induced apoptosis in cultured mouse oligodendrocyte progenitor cells using primarily the PI3K/Akt pathway. Mol Cell Neurosci.

[B200] Zeger M, Popken G, Zhang J, Xuan S, Lu QR, Schwab MH, Nave KA, Rowitch D, D’Ercole AJ, Ye P (2007). Insulin-like growth factor type 1 receptor signaling in the cells of oligodendrocyte lineage is required for normal *in vivo* oligodendrocyte development and myelination. Glia.

[B201] Zhou Q, Anderson DJ (2002). The bHLH transcription factors OLIG2 and OLIG1 couple neuronal and glial subtype specification. Cell.

[B202] Zolova OE, Wight PA (2011). YY1 negatively regulates mouse myelin proteolipid protein (Plp1) gene expression in oligodendroglial cells. ASN NEURO.

[B203] Zou J, Zhou L, Du XX, Ji Y, Xu J, Tian J, Jiang W, Zou Y, Yu S, Gan L, Luo M, Yang Q, Cui Y, Yang W, Xia X, Chen M, Zhao X, Shen Y, Chen PY, Worley PF, Xiao B (2011). Rheb1 is required for mTORC1 and myelination in postnatal brain development. Dev Cell.

